# Controlling spin supercurrents via nonequilibrium spin injection

**DOI:** 10.1038/s41598-019-48945-0

**Published:** 2019-09-13

**Authors:** Jabir Ali Ouassou, Jason W. A. Robinson, Jacob Linder

**Affiliations:** 1 Center for Quantum Spintronics, Department of Physics, Norwegian University of Science and Technology, NO-7491 Trondheim, Norway; 2 Department of Materials Science and Metallurgy, University of Cambridge, 27 Charles Babbage Road, Cambridge, CB3 0FS United Kingdom

**Keywords:** Spintronics, Superconducting properties and materials

## Abstract

We propose a mechanism whereby spin supercurrents can be manipulated in superconductor/ferromagnet proximity systems via nonequilibrium spin injection. We find that if a spin supercurrent exists in equilibrium, a nonequilibrium spin accumulation will exert a torque on the spins transported by this current. This interaction causes a new spin supercurrent contribution to manifest out of equilibrium, which is proportional to and polarized perpendicularly to both the injected spins and the equilibrium spin current. This is interesting for several reasons: as a fundamental physical effect; due to possible applications as a way to control spin supercurrents; and timeliness in light of recent experiments on spin injection in proximitized superconductors.

## Introduction

In the field of superconducting spintronics, a key objective is to study the interactions between superconductors (S) and ferromagnets (F)^1–4^. These interactions produce new types of Cooper pairs |↑↑〉 and |↓↓〉 with a net spin polarization, enabling the use of S/F systems for dissipationless spin transport. From a fundamental physics perspective, such an interplay between different types of quantum order is expected to give rise to interesting physics to explore. Ultimately, the ambition is to exploit these phenomena in devices related to e.g. supercomputing and ultrasensitive detection of heat and radiation.

One particular area that has attracted attention is how one may generate controllable spin supercurrents in equilibrium, either via inhomogeneous magnetism^5–10^ or spin-orbit coupling^11–14^. In the magnetic case, it has been shown that two layers with noncollinear magnetic moments ***m***_1_ and ***m***_2_ give rise to an equilibrium spin supercurrent ${{\boldsymbol{j}}}_{{\rm{s}}}^{{\rm{eq}}} \sim {{\boldsymbol{m}}}_{1}\times {{\boldsymbol{m}}}_{2}$^15^. While most work so far relies on magnetic control of spin supercurrents via rotation of ***m***_1_ relative to ***m***_2_, it would be interesting to determine if a spin supercurrent can be controlled via electronic spin injection instead. Such a mechanism might be more beneficial for coupling superconducting and nonsuperconducting spintronics devices. Note that this is different from many previous works on spin injection in superconductors, which were largely explained in terms of quasiparticles and not a spin-triplet condensate^16–22^.

Recently, there has been a renewed interest in using nonequilibrium spin injection as a means to manipulate spin supercurrents. This is largely due to a recent spin-pumping experiment^23^, where microwaves were used to excite spins in the ferromagnetic layer of an N/S/F/S/N junction. This experiment showed that the pumped spin current could increase below the critical temperature *T*_c_ of the S layers. It has been proposed that this effect was assisted by a Cooper-pair spin supercurrent^13^, although alternative explanations have been proposed^24^.

**Figure 1:**
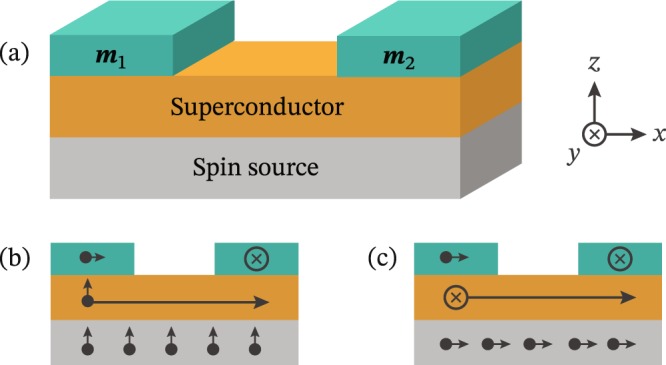
(**a**) Magnetic insulators with magnetizations ***m***_1_ and ***m***_2_ on a superconductor. In equilibrium, this yields a spin supercurrent ${{\boldsymbol{J}}}_{{\rm{s}}}^{{\rm{eq}}} \sim {{\boldsymbol{m}}}_{1}\times {{\boldsymbol{m}}}_{2}$. A spin source injects a spin accumulation ***ρ***_s_, which exerts a torque on the spins transported by the equilibrium current, resulting in a new contribution ${{\boldsymbol{J}}}_{{\rm{s}}}^{{\rm{neq}}} \sim {{\boldsymbol{\rho }}}_{{\rm{s}}}\times ({{\boldsymbol{m}}}_{1}\times {{\boldsymbol{m}}}_{2})$. (**b**) If the magnets are magnetized in the *x*- and *y*-directions, an equilibrium spin-*z* supercurrent arises. Injection of spin-*z* particles does not affect its polarization. Note that a spin supercurrent is in general a rank-2 tensor, encoding both a polarization (short arrow) and transport direction (long arrow). (**c**) If spin-*x* particles are injected, however, a new spin-*y* supercurrent component is generated. Similarly, spin-*y* injection would produce a spin-*x* component. We model this setup as a 1D system, where the magnetic insulators connect to the superconductor at the sides; but in the diffusive limit, this should yield physically equivalent results to the setup depicted in this figure.

In this manuscript, we consider how an injected nonequilibrium spin accumulation in general affects an existing spin supercurrent (see Fig. 1). We show that such a spin injection actually produces new terms in the equations for the spin supercurrent itself. These terms have a natural interpretation in the form of the injected spin accumulation ***ρ***_s_ exerting a torque on an equilibrium spin supercurrent ${{\boldsymbol{j}}}_{{\rm{s}}}^{{\rm{eq}}}$, thus giving rise to a new component ${{\boldsymbol{J}}}_{{\rm{s}}}^{{\rm{neq}}} \sim {{\boldsymbol{\rho }}}_{{\rm{s}}}\times {{\boldsymbol{J}}}_{{\rm{s}}}^{{\rm{eq}}}$ perpendicular to both. Although this term occurs out-of-equilibrium, it shares the property of an equilibrium spin supercurrent that it does not require gradients in any chemical potential. Therefore, it is legitimate to refer to the new term ${{\boldsymbol{j}}}_{{\rm{s}}}^{{\rm{neq}}}$ as a supercurrent flowing without dissipation, as there is no energy loss associated with a spatially varying chemical potential. Our result is different from e.g. ref. 13, which proposed that *equilibrium* spin accumulation might produce spin supercurrents in some materials.

## Results

### Analytical results

Let us first consider a material with a spin-independent density of states *N*(*ε*), where *ε* is the quasiparticle energy. The nonequilibrium spin accumulation ***ρ***_s_ can then be related to a spin distribution function ***h***_s_,

1 $${{\boldsymbol{\rho }}}_{{\rm{s}}}=-\frac{\hslash }{2}{\int }_{0}^{\infty }{\rm{d}}\,\epsilon \,N(\epsilon ){{\boldsymbol{h}}}_{{\rm{s}}}(\epsilon ),$$

where ***h***_s_ describes the imbalance between spin-up and spin-down occupation numbers. We define ***h***_s_ as a vector that points in the polarization direction of the spins, while its magnitude can be described in terms of a *spin voltage V*_s_,

2 $${h}_{{\rm{s}}}(\epsilon )=\{\,\tanh \,[(\epsilon +e{V}_{{\rm{s}}}\mathrm{)/2}T]-\,\tanh \,[(\epsilon -e{V}_{{\rm{s}}}\mathrm{)/2}T]\,\mathrm{\}/2,}$$

where *e* < 0 is the electron charge and *T* is the temperature. The spin voltage is defined as *V*_s_ := (*V*_↑_ − *V*_↓_)/2, where *V*_*σ*_ are the effective potentials seen by spin-*σ* quasiparticles^25–27^, and ***h***_s_ defines the spin-quantization axis.

For a normal metal at *T* = 0, the density of states *N*(*∊*) = *N*_0_ is flat, while the spin distribution |***h***_s_| = 1 for |*∊*| < *eV*_s_. This results in a spin accumulation |***ρ***_s_| = (*ħ*/2)*N*_0_*eV*_s_ that increases linearly with *V*_s_. This gives a simple interpretation of *V*_s_ as a control parameter: if the spin source in Fig. 1 is a nonsuperconducting reservoir, then the spin voltage *V*_s_ is directly proportional to the spin accumulation in the reservoir.

Similarly to the above, the excitation of quasiparticles from the Fermi level is decribed by an energy distribution *h*_0_(*∊*),

3 $${h}_{{\rm{0}}}(\epsilon )=\{\,\tanh \,[(\epsilon +e{V}_{{\rm{s}}}\mathrm{)/2}T]+\,\tanh \,[(\epsilon -e{V}_{{\rm{s}}}\mathrm{)/2}T]\,\mathrm{\}/2.}$$

At low temperatures, this shows that a spin voltage *V*_s_ also excites quasiparticles in a region of width 2*eV*_s_ around the Fermi level *∊* = 0. For a more in-depth discussion of the nonequilibrium distribution function, see refs ^20,25,26^.

Spin supercurrents can in general be expressed as energy integrals of spectral spin supercurrents,

4 $${{\boldsymbol{J}}}_{{\rm{s}}}=-\frac{\hslash }{2}{N}_{0}{\int }_{0}^{\infty }{\rm{d}}\,\epsilon \,\mathrm{Im}[\,{{\boldsymbol{j}}}_{{\rm{s}}}]{\rm{.}}$$

In equilibrium, the spectral current ${{\boldsymbol{j}}}_{{\rm{s}}}^{{\rm{eq}}}$ is given by^3,10^

5 $${{\boldsymbol{j}}}_{{\rm{s}}}^{{\rm{eq}}}=({{\boldsymbol{g}}}_{t}\times \nabla {{\boldsymbol{g}}}_{t}-{{\boldsymbol{f}}}_{t}\times \nabla {\tilde{{\boldsymbol{f}}}}_{t}){h}_{0}\mathrm{.}$$

Here, ***g***_*t*_ describes the spin-polarization of the density of states, while ***f***_*t*_ describes spin-triplet correlations^3^. The cross products should be taken between the orientations of the vectors ***g***_*t*_ and ***f***_*t*_. Since the gradient of a vector is a rank-2 tensor, the spin supercurrent is such a tensor, enabling it to encode both the spin polarization and the transport direction. However, in effectively 1D systems like Fig. 1, we can let the position derivative ▽ → ∂_*x*_. In other words, in systems with 1D transport, the spin supercurrent reduces to a vector that describes spin polarization. Note that the result depends only on the energy distribution *h*_0_, which is the only part of the distribution function which remains finite in equilibrium.

Outside equilibrium, the spin distribution ***h***_s_ can become finite, and the spectral current gains an additional contribution:

6 $${{\boldsymbol{j}}}_{{\rm{s}}}^{{\rm{neq}}}=({{\boldsymbol{g}}}_{t}\times \nabla {{\boldsymbol{g}}}_{t}-{{\boldsymbol{f}}}_{t}\times \nabla {\tilde{{\boldsymbol{f}}}}_{t})\times i{{\boldsymbol{h}}}_{{\rm{s}}}.$$

The full derivation of this result is included in the Supplementary Information (Sec. II). The structure of Eq. (6) is very reminiscent of Eq. (5), since both depend on ${{\boldsymbol{g}}}_{t}\times \nabla {{\boldsymbol{g}}}_{t}-{{\boldsymbol{f}}}_{t}\times \nabla {\tilde{{\boldsymbol{f}}}}_{t}$. However, its cross product structure generates a spin current *perpendicular* to the one in Eq. (5). We also see that it contains an extra factor *i*; since the distribution functions *h*_0_ and ***h***_s_ are both real, this causes Eq. (4) to extract the real and not imaginary part of ${{\boldsymbol{g}}}_{t}\times \nabla {{\boldsymbol{g}}}_{t}-{{\boldsymbol{f}}}_{t}\times \nabla {\tilde{{\boldsymbol{f}}}}_{t}$. This comparison shows that the nonequilibrium contribution can be summarized as

7 $${{\boldsymbol{j}}}_{{\rm{s}}}^{{\rm{neq}}}={{\boldsymbol{j}}}_{{\rm{s}}}^{{\rm{eq}}}\times (i{{\boldsymbol{h}}}_{{\rm{s}}}/{h}_{0}\mathrm{).}$$

So long as ***g***_*t*_ × ▽***g***_*t*_ − ***f***_*t*_ × ▽***f***_*t*_ is a complex number—which it in general is—it produces an equilibrium spin supercurrent ${{\boldsymbol{j}}}_{{\rm{s}}}^{{\rm{eq}}}$ according to Eqs (4) and (5), which combined with a finite spin distribution ***h***_s_ immediately produces the new supercurrent term ${{\boldsymbol{j}}}_{{\rm{s}}}^{{\rm{neq}}}$ according to Eq. (7). This suggests an intuitive interpretation of the effect: injected spins described by ***h***_s_ exert a torque on the spins transported by the equilibrium component ${{\boldsymbol{j}}}_{{\rm{s}}}^{{\rm{eq}}}$, producing a nonequilibrium component ${{\boldsymbol{j}}}_{{\rm{s}}}^{{\rm{neq}}}$ perpendicular to both. It also suggests that the nonequilibrium spin supercurrent should increase linearly with the equilibrium spin supercurrent and the injected spin accumulation. Thus, an equilibrium spin supercurrent gains a new component when propagating through a region with spin accumulation ***ρ***_s_. All these predictions that arise from Eq. (7) are confirmed numerically later in this paper.

Let us now consider the setup in Fig. 1. In equilibrium, the *x*- and *y*-polarized magnets give rise to a *z*-polarized spin supercurrent ${{\boldsymbol{j}}}_{{\rm{s}}}^{{\rm{eq}}} \sim {\bf{z}}$. A generic spin source then introduces a spin imbalance in the superconductor, which we describe via a nonzero spin distribution ***h***_s_. If these spins are polarized in the *z*-direction, meaning that ${{\boldsymbol{h}}}_{{\rm{s}}}\,\parallel \,{{\boldsymbol{j}}}_{{\rm{s}}}^{{\rm{eq}}}$, then the nonequilibrium contribution ${{\boldsymbol{j}}}_{{\rm{s}}}^{{\rm{neq}}}=0$. On the other hand, if these spins are polarized in the *x*-direction, so that ${{\boldsymbol{h}}}_{{\rm{s}}}\perp {{\boldsymbol{j}}}_{{\rm{s}}}^{{\rm{eq}}}$, then the nonequilibrium contribution ${{\boldsymbol{j}}}_{{\rm{s}}}^{{\rm{neq}}} \sim {{\boldsymbol{j}}}_{{\rm{s}}}^{{\rm{eq}}}\times {{\boldsymbol{h}}}_{{\rm{s}}}$ obtains a *y*-polarized component proportional to the spin imbalance. Similarly, if one had injected spin-*y* particles instead, a spin-*x* supercurrent would appear in the superconductor. In our calculations, we will for simplicity use an effective 1D model where the magnetic insulators are connected to the superconducting region at its sides rather than deposited on top of the superconductor. In practice, this has essentially no consequence since we are considering the diffusive limit of transport. The reason for this is that the spin supercurrent flow through the superconductor arises regardless of the exact spatial point at the edge of the superconductor (on top or at its side) where the magnetic insulators couple to the Cooper pairs, and in the diffusive limit the randomized motion of charge carriers further makes the precise coupling point irrelevant. Thus, our calculations should correspond well to the suggested setup in Fig. 1.

To summarize, for the geometry in Fig. 1, the analytical results suggest that we should expect a spin-*y* supercurrent proportional to the spin-*x* voltage, while the spin supercurrent should remain unchanged for a spin-*z* voltage. In the following sections, we compare these expectations to numerical results.

### Numerical results

The spin supercurrent in the model considered here is conserved throughout the superconductor. In fact, we have checked both analytically and numerically that the spin supercurrent remains conserved in the presence of spin-flip and spin-orbit impurities, thus extending the equilibrium results from ref. 28 to this particular nonequilibrium situation. The analytical proof is straight-forward: the argument in ref. 28 shows that $\nabla \cdot {{\boldsymbol{j}}}_{{\rm{s}}}^{{\rm{eq}}}\,=\,0$ as long as *h*_0_ is position-independent. Since the new contribution proposed in this paper ${{\boldsymbol{j}}}_{{\rm{s}}}^{{\rm{neq}}}={{\boldsymbol{j}}}_{{\rm{s}}}^{{\rm{eq}}}\times (i{{\boldsymbol{h}}}_{{\rm{s}}}/{h}_{0})$, we conclude that $\nabla \cdot {{\boldsymbol{j}}}_{{\rm{s}}}^{{\rm{neq}}}=0$ if ***h***_s_ is position-independent. However, if either *h*_0_ or ***h***_s_ becomes inhomogeneous, this argument breaks down, and the spin supercurrent is no longer conserved.

**Figure 2:**
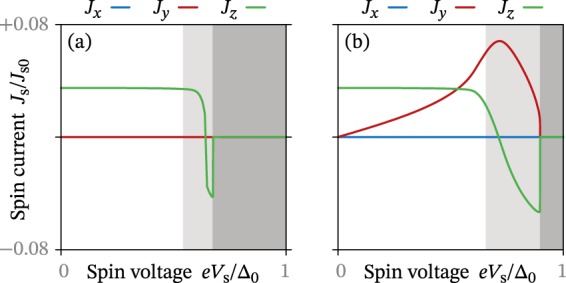
Spin supercurrent ***J***_s_ as a function of spin voltage *V*_s_. The spin voltage corresponds to injected (**a**) spin-*z* or (**b**) spin-*x* accumulation. The light shaded regions show where the system is bistable, and the dark ones where superconductivity vanishes.

In Fig. 2, we show the spin supercurrent in the superconductor as a function of spin voltage at a low temperature *T* = 0.01*T*_c_. Up until *eV*_s_ ≈ Δ_0_/2, where Δ_0_ is the bulk superconducting gap at zero temperature, these results are in perfect agreement with the analytical predictions. More precisely, we see that a spin-*z* injection (Fig. 2(a)) has no effect on the spin supercurrent, while a spin-*x* injection (Fig. 2(b)) leads to a spin-*y* supercurrent. The spin-*y* supercurrent increases linearly with the spin voltage, again in agreement with the predictions. Remarkably, the spin-*z* supercurrent does not decrease as the spin-*y* supercurrent increases, in contrast to what one might intuitively expect.

At low temperatures, we also see that there is a bistable regime at high spin voltages *eV*_s_ > Δ_0_/2. This means that both a superconducting and normal-state solution exist, which both correspond to local minima in the free energy. Depending on the dynamics of the system, this can either lead to hysteretic behaviour, or a first-order phase transition. This first-order phase transition was discussed already in the 1960s by Chandrasekhar and Clogston^29,30^, while the possibility of hysteretic behaviour was suggested more recently in refs 31,32. Precisely where in the bistable region the thermodynamic transition point occurs is however difficult to predict within the Usadel formalism, as it is not straight-forward to explicitly evaluate the free energy^25^.

Within the bistable regime, there is a point where the spin-*z* supercurrent reverses direction as a function of the spin voltage. This behaviour can be understood^33^ as a spin equivalent of the S/N/S transistor effect^34,35^ where, according to Eq. (3), the energy distribution *h*_0_ is also modulated by a spin voltage, and may therefore tune the equilibrium contribution in Eq. (5).

Since the spin-*y* supercurrent remains positive for all spin voltages, there exists a point where we get a pure spin-*y* supercurrent. In other words, there is a particular spin voltage that causes a 90° rotation of the spin supercurrent polarization compared to equilibrium. The fact that the spin-*y* supercurrent can remain finite while the spin-*z* supercurrent goes to zero might at first seem contradictory to our previous explanation ${{\boldsymbol{j}}}_{{\rm{s}}}^{{\rm{neq}}} \sim {{\boldsymbol{j}}}_{{\rm{s}}}^{{\rm{eq}}}\times (i{{\boldsymbol{h}}}_{{\rm{s}}}/{h}_{0})$. However, it is the *energy-integrated* spin currents ${{\boldsymbol{J}}}_{{\rm{s}}} \sim \int {\rm{d}}\epsilon \,{\rm{Im}}[\,{{\boldsymbol{j}}}_{{\rm{s}}}]$ that are plotted in Fig. 2. The spin-*y* current is generated from the *spectral* spin-*z* current, which remains finite even though the *total* spin-*z* current is zero.

**Figure 3:**
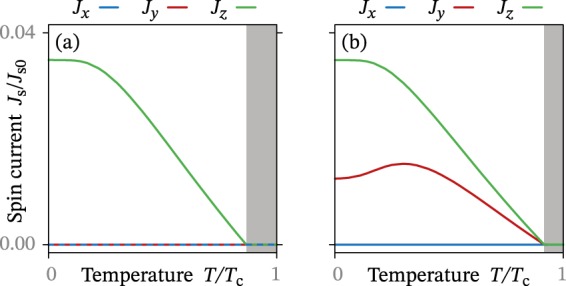
Spin supercurrent ***J***_s_ as a function of temperature *T* for a fixed spin voltage *eV*_s_ = Δ_0_/4 in the superconductor. This spin voltage corresponds to injected (**a**) spin-*z* or (**b**) spin-*x* accumulation. Superconductivity vanishes in the shaded region.

In Fig. 3, we show how the spin supercurrent varies as a function of temperature for a fixed spin voltage *eV*_s_ = Δ_0_/4. Curiously, we find that the spin current increases *linearly* with decreasing temperature in a relatively large parameter regime. That the spin-*y* current decreases at the same rate as the spin-*z* current seems reasonable in light of the equation ${{\boldsymbol{j}}}_{{\rm{s}}}^{{\rm{neq}}} \sim {{\boldsymbol{j}}}_{{\rm{s}}}^{{\rm{eq}}}\times {{\boldsymbol{h}}}_{{\rm{s}}}$: if ${{\boldsymbol{j}}}_{{\rm{s}}}^{{\rm{eq}}}$ decreases linearly, then ${{\boldsymbol{j}}}_{{\rm{s}}}^{{\rm{neq}}}$ should do so as well. The most important message from Fig. 3 is perhaps that the nonequilibrium contribution ${{\boldsymbol{j}}}_{{\rm{s}}}^{{\rm{neq}}}$ to the spin supercurrent remains significant all the way up to the critical temperature of the junction. This means that relevant experiments can be performed at any temperature where superconductivity exists.

## Discussion

In the previous sections, we have shown that injection of a nonequilibrium spin accumulation can be used to generate new spin supercurrent components. The results are especially encouraging since the nonequilibrium contribution to the spin supercurrent can even be made larger than the equilibrium contribution, and we found that it persists all the way up to the critical temperature of the junction. Both these features should make it a particularly interesting effect for experimental detection and future device design. However, there are some questions that we have not addressed yet.

The first question is how the spin source in Fig. 1 works. So far, we have simply treated it as a generic device that manipulates the spin distribution ***h***_s_ inside the superconductor directly. One alternative is to use a normal metal coupled to a voltage-biased ferromagnet^18^ or half-metallic ferromagnet^36^. In that case, the polarization of the magnets enable a charge-spin conversion, thus translating an electric voltage into a spin voltage. Another possibility would be spin-pumping experiments, where it is a microwave signal that is translated to a spin voltage^23^. In the limit of weak superconductivity, an expression for the distribution function of a spin-pumped ferromagnet was derived in ref. 37. When the precession frequency Ω and cone angle *α* are sufficiently small, the result is just a spin voltage *eV*_s_ = Ω/2 along the magnetization direction of the ferromagnet. This is the relevant limit for the experiment in ref. 23: superconductivity inside Ni_80_Fe_20_ is weak, Ω ≈ 0.5 meV is much smaller than its magnetic exchange field, and *α* ≈ 1° should be small enough to use the leading-order expansions sin*α* ≈ *α* and cos*α* ≈ 1.

In all these cases, the spin source necessarily contains magnetic elements, and one challenge would be how to prevent the spin source from affecting the *equilibrium* spin current. One solution might be to embrace the existing magnets in Fig. 1: one could use the same magnets to generate the equilibrium spin supercurrent and for spin injection. This spin injection may them be performed either using spin pumping—or if the magnets are sufficiently thin for electron tunneling—by placing voltage-biased contacts on top of the magnets. One complication with this strategy is that since the resulting spin accumulation will necessarily be inhomogeneous, both spin supercurrents and resistive spin currents have to coexist.

How to directly measure a spin supercurrent is an open question, although suggestions have recently been proposed^38^. Indirect measurements of spin supercurrents, on the other hand, have already been performed experimentally. Most of these rely on measuring dissipationless charge currents through strongly polarized materials^39–46^. Since only |↑↑〉 and |↓↓〉 pairs can penetrate over longer distances, and the polarization breaks the degeneracy between them, one can infer the existence of spin supercurrents from the measured charge supercurrents.

One solution to the measurement problem might be to look for an *inverse* effect. We have shown that spin injection into a superconductor results in a torque on the spins transported by the equilibrium spin supercurrent. However, this interaction should cause a reaction torque on the spin source, which might be possible to detect. For instance, in a setup similar to ref. 18, this reaction torque might directly affect the nonlocal spin conductance. Similarly, in a spin-pumping setup, this might affect the FMR linewidths. In both cases, this reaction torque should only exist when there is an equilibrium spin supercurrent ${{\boldsymbol{j}}}_{{\rm{s}}}^{{\rm{eq}}} \sim {{\boldsymbol{m}}}_{1}\times {{\boldsymbol{m}}}_{2}$ to interact with, so it should depend on the magnetic configuration of the device.

We have shown analytically and numerically that if a system harbors a spin supercurrent ${{\boldsymbol{j}}}_{{\rm{s}}}^{{\rm{eq}}}$ in equilibrium, then a spin injection ***h***_s_ creates a new component ${{\boldsymbol{j}}}_{{\rm{s}}}^{{\rm{neq}}} \sim {{\boldsymbol{j}}}_{{\rm{s}}}^{{\rm{eq}}}\times {{\boldsymbol{h}}}_{{\rm{s}}}$. This effect can be intuitively understood as the injected spins exerting a torque on the spins transported by the equilibrium spin supercurrent, generating a component that is perpendicular to both. These results have implications for the control of spin supercurrents in novel superconducting spintronics devices.

## Methods

In this section, we briefly describe how our numerical results were obtained. For more details about the numerical model, see the Supplementary Information (Sec. III). The numerical implementation is available at https://github.com/jabirali/GENEUS.

The numerical calculations were performed using the Usadel equation^26,47–50^, which provides a good description of superconducting systems in the quasiclassical and diffusive limits. Within this formalism, physical observables are described via 8 × 8 quasiclassical propagators in Keldysh ⊗ Nambu ⊗ spin space,

8 $$\check{g}\,:=(\begin{array}{cc}{\hat{g}}^{{\rm{R}}} & {\hat{g}}^{{\rm{K}}}\\ 0 & {\hat{g}}^{{\rm{A}}}\end{array})\mathrm{.}$$

These matrix components are related by the identities ${\hat{g}}^{{\rm{K}}}={\hat{g}}^{{\rm{R}}}\hat{h}-\hat{h}{\hat{g}}^{{\rm{A}}}$ and ${\hat{g}}^{{\rm{A}}}={\hat{\tau }}_{3}\,{\hat{g}}^{{\rm{R}}\dagger }{\hat{\tau }}_{3}$. Here $\hat{h}$, is a 4 × 4 distribution function, which in systems with spin accumulation can be written

9 $$\hat{h}={h}_{0}{\hat{\sigma }}_{0}{\hat{\tau }}_{0}+{{\boldsymbol{h}}}_{{\rm{s}}}\cdot \hat{{\boldsymbol{\sigma }}}{\hat{\tau }}_{3},$$

where *h*_0_ and ***h***_s_ were introduced earlier. The ${\hat{\tau }}_{n}$ are ${\hat{\sigma }}_{n}$ are Pauli matrices in Nambu and spin space, respectively. As for the retarded component ${\hat{g}}^{{\rm{R}}}$, we analytically use the parametrization^3^

10 $${\hat{g}}^{{\rm{R}}}=(\begin{array}{cc}(\,{g}_{s}+{{\boldsymbol{g}}}_{t}\cdot {\boldsymbol{\sigma }}) & (\,{f}_{s}+{{\boldsymbol{f}}}_{t}\cdot {\boldsymbol{\sigma }})i{\sigma }_{2}\\ -i{\sigma }_{2}(\,{\tilde{f}}_{s}-{\tilde{{\boldsymbol{f}}}}_{t}\cdot {\boldsymbol{\sigma }}) & -{\sigma }_{2}(\,{\tilde{g}}_{s}-{\tilde{{\boldsymbol{g}}}}_{t}\cdot {\boldsymbol{\sigma }}){\sigma }_{2}\end{array}),$$

while we numerically use the Riccati parametrization^51^. General equations for calculating spin supercurrents and spin accumulations from these quasiclassical propagators are derived and presented in the Supplementary Information (Sec. I).

To determine the propagators above for the setup in Fig. 1, we have to simultaneously solve the Usadel equation,

11 $$i{\xi }^{2}\nabla (\check{g}\nabla \check{g})=[\hat{\Delta }+\epsilon {\hat{\tau }}_{3},\,\check{g}]/{\Delta }_{0},$$

and a selfconsistency equation for the gap Δ which depends on ${\hat{g}}^{{\rm{K}}}$^52^. The other quantities are the dirty-limit coherence length *ξ* and bulk gap Δ_0_. The magnetic insulators in Fig. 1 are modelled as spin-active interfaces^53–56^.

We assume a fixed distribution function $\hat{h}$, and do not solve any kinetic equation^20,21,25,26,47,48,57^. This approximation is valid when the superconducting layer is thin compared to its spin relaxation length. Thus, there is no resistive spin current flowing in the superconductor, as ▽***h***_s_ = 0 ensures that there is no gradient in the spin accumulation.

Finally, we briefly summarize our parameter choices. The superconductor was taken to have a length *L* = 1.5*ξ*. The magnetic insulators were described with *G*_*φ*_/*G*_N_ = 0.6, where *G*_N_ is the bulk normal-state conductance of the superconductor, and *G*_*φ*_ describes the spin-dependent phase-shifts obtained by quasiparticles reflected at a magnetic interface^53–56^. Finally, we assumed a constant spin voltage *V*_s_ throughout the entire superconductor, instead of explicitly modelling the details of the spin source in Fig. 1. Thus, the junction is treated as a 1D superconductor with magnetic boundary conditions. Our results are not qualitatively sensitive to these parameter choices. The main constraints are that superconductivity collapses if *L*/*ξ* is too low and *G*_*φ*_/*G*_N_ too high, while spin supercurrents become vanishingly small in the opposite limits.

## Supplementary information

Supplementary Information

## References

[1] Linder J, Robinson JWA (2015). Superconducting spintronics. Nature Physics.

[2] Eschrig M (2010). Spin-polarized supercurrents for spintronics. Physics Today.

[3] Eschrig M (2015). Spin-polarized supercurrents for spintronics. Reports on Progress in Physics.

[4] Blamire MG, Robinson JWA (2014). The interface between superconductivity and magnetism. Journal of Physics: Condensed matter.

[5] Grein R, Eschrig M, Metalidis G, Schön G (2009). Spin-dependent Cooper pair phase and pure spin supercurrents in strongly polarized ferromagnets. Physical Review Letters.

[6] Alidoust M, Linder J, Rashedi G, Yokoyama T, Sudbø A (2010). Spin-polarized Josephson current in superconductor/ferromagnet/superconductor junctions with inhomogeneous magnetization. Physical Review B.

[7] Shomali Z, Zareyan M, Belzig W (2011). Spin supercurrent in Josephson contacts with noncollinear ferromagnets. New Journal of Physics.

[8] Brydon PMR, Asano Y, Timm C (2011). Spin Josephson effect with a single superconductor. Physical Review B.

[9] Moor A, Volkov AF, Efetov KB (2015). Nematic versus ferromagnetic spin filtering of triplet Cooper pairs in superconducting spintronics. Physical Review B.

[10] Gomperud I, Linder J (2015). Spin supercurrent and phase-tunable triplet Cooper pairs via magnetic insulators. Physical Review B.

[11] Konschelle F, Tokatly IV, Bergeret FS (2015). Theory of the spin-galvanic effect and the anomalous phase shift *ϕ*_0_ in superconductors and Josephson junctions with intrinsic spin–orbit coupling. Physical Review B.

[12] Jacobsen SH, Kulagina I, Linder J (2016). Controlling superconducting spin flow with spin-flip immunity using a single homogeneous ferromagnet. Scientific Reports.

[13] Montiel X, Eschrig M (2018). Generation of pure superconducting spin current in magnetic heterostructures via nonlocally induced magnetism due to Landau Fermi liquid effects. Physical Review B.

[14] Bobkova IV, Barash YS (2004). Effects of spin–orbit interaction on superconductor–ferromagnet heterostructures. JETP Letters.

[15] Slonczewski JC (1989). Conductance and exchange coupling of two ferromagnets separated by a tunneling barrier. Physical Review B.

[16] Beckmann D (2016). Spin manipulation in nanoscale superconductors. Journal of Physics: Condensed Matter.

[17] Quay CHL, Chevallier D, Bena C, Aprili M (2013). Spin imbalance and spin–charge separation in a mesoscopic superconductor. Nature Physics.

[18] Wakamura T, Hasegawa N, Ohnishi K, Niimi Y, Otani Y (2014). Spin injection into a superconductor with strong spin–orbit coupling. Physical Review Letters.

[19] Hübler F, Wolf MJ, Beckmann D, v. Löhneysen H (2012). Long-range spin-polarized quasiparticle transport in mesoscopic Al superconductors with a Zeeman splitting. Physical Review Letters.

[20] Silaev M, Virtanen P, Bergeret FS, Heikkilä TT (2015). Long-range spin accumulation from heat injection in mesoscopic superconductors with Zeeman splitting. Physical Review Letters.

[21] Bobkova IV, Bobkov AM (2015). Recovering of superconductivity in S/F bilayers under spin-dependent nonequilibrium quasiparticle distribution. JETP letters.

[22] Bobkova IV, Bobkov AM (2016). Injection of nonequilibrium quasiparticles into Zeeman-split superconductors. Physical Review B.

[23] Jeon K-R (2018). Enhanced spin pumping into superconductors provides evidence for superconducting pure spin currents. Nature Materials.

[24] Taira, T., Ichioka, M., Takei, S. & Adachi, H. Spin diffusion equation in superconductors in the vicinity of *T*_*c*_. *Physical Review B* **98** (2018).

[25] Ouassou JA, Vethaak TD, Linder J (2018). Voltage-induced thin-film superconductivity in high magnetic fields. Physical Review B.

[26] Bergeret FS, Silaev M, Virtanen P, Heikkilä TT (2018). Colloquium: Nonequilibrium effects in superconductors with a spin-splitting field. Reviews of Modern Physics.

[27] Bauer GEW, Saitoh E, van Wees BJ (2012). Spin caloritronics. Nature Materials.

[28] Ouassou JA, Jacobsen SH, Linder J (2017). Conservation of spin supercurrents in superconductors. Physical Review B.

[29] Chandrasekhar BS (1962). A note on the maximal critical field of high-field superconductors. Applied Physics Letters.

[30] Clogston AM (1962). Upper limit for the critical field in hard superconductors. Physical Review Letters.

[31] Bobkova IV, Bobkov AM (2014). Bistable state in superconductor/ferromagnet heterostructures. Physical Review B.

[32] Snyman I, Nazarov YV (2009). Bistability in voltage-biased normal-metal/insulator/superconductor/insulator/normal-metal structures. Physical Review B.

[33] Ouassou, J.A. & Linder, J. Voltage control of superconducting exchange interaction and anomalous Josephson effect. Preprint at http://arxiv.org/abs/1810.02820 (2018).

[34] Wilhelm FK, Schön G, Zaikin AD (1998). Mesoscopic superconducting–normal metal–superconducting transistor. Physical Review Letters.

[35] Baselmans JJA, Morpurgo AF, van Wees BJ, Klapwijk TM (1999). Reversing the direction of the supercurrent in a controllable Josephson junction. Nature.

[36] Bobkova IV, Bobkov AM (2012). Long-range proximity effect for opposite-spin pairs in superconductor–ferromagnet heterostructures under nonequilibrium quasiparticle distribution. Physical Review Letters.

[37] Houzet M (2008). Ferromagnetic Josephson junction with precessing magnetization. Physical Review Letters.

[38] Risinggård, V. & Linder, J. Direct and inverse superspin Hall effect in two-dimensional systems. Preprint at http://arxiv.org/abs/1902.05555 (2019).

[39] Keizer RS (2006). A spin triplet supercurrent through the half-metallic ferromagnet CrO_2_. Nature.

[40] Anwar MS, Czeschka F, Hesselberth M, Porcu M, Aarts J (2010). Long-range supercurrents through half-metallic ferromagnetic CrO_2_. Physical Review B.

[41] Robinson JWA, Witt JDS, Blamire MG (2010). Controlled injection of spin-triplet supercurrents into a strong ferromagnet. Science.

[42] Robinson JWA, Halász GB, Buzdin AI, Blamire MG (2010). Enhanced supercurrents in Josephson junctions containing nonparallel ferromagnetic domains. Physical Review Letters.

[43] Khaire TS, Khasawneh MA, Pratt WP, Birge NO (2010). Observation of spin-triplet superconductivity in Co-based Josephson junctions. Physical Review Letters.

[44] Witt JDS, Robinson JWA, Blamire MG (2012). Josephson junctions incorporating a conical magnetic holmium interlayer. Physical Review B.

[45] Robinson JWA, Banerjee N, Blamire MG (2014). Triplet pair correlations and nonmonotonic supercurrent decay with Cr thickness in Nb/Cr/Fe/Nb Josephson devices. Physical Review B.

[46] Egilmez M (2014). Supercurrents in half-metallic ferromagnetic La_0.7_Ca_0.3_MnO_3_. Europhysics Letters.

[47] Chandrasekhar, V. Proximity-coupled systems in *Superconductivity: Conventional and Unconventional Superconductors* (eds Bennemann, K. H. & Ketterson, J. B.) 279–313 (Springer, 2008).

[48] Belzig W, Wilhelm FK, Bruder C, Schön G, Zaikin AD (1999). Quasiclassical Green’s function approach to mesoscopic superconductivity. Superlattices and Microstructures.

[49] Rammer J, Smith H (1986). Quantum field-theoretical methods in transport theory of metals. Reviews of Modern Physics.

[50] Usadel KD (1970). Generalized diffusion equation for superconducting alloys. Physical Review Letters.

[51] Schopohl, N. Transformation of the Eilenberger equations of superconductivity to a scalar Riccati equation. Preprint at https://arxiv.org/abs/cond-mat/9804064 (1998).

[52] Jacobsen SH, Ouassou JA, Linder J (2015). Critical temperature and tunneling spectroscopy of superconductor–ferromagnet hybrids with intrinsic Rashba–Dresselhaus spin–orbit coupling. Physical Review B.

[53] Eschrig M, Cottet A, Belzig W, Linder J (2015). General boundary conditions for quasiclassical theory of superconductivity in the diffusive limit. New Journal of Physics.

[54] Machon P, Eschrig M, Belzig W (2013). Nonlocal thermoelectric effects and nonlocal Onsager relations in a three-terminal proximity-coupled superconductor–ferromagnet device. Physical Review Letters.

[55] Cottet A, Huertas-Hernando D, Belzig W, Nazarov YV (2009). Spin-dependent boundary conditions for isotropic superconducting Green’s functions. Physical Review B.

[56] Cottet A (2007). Spectroscopy and critical temperature of diffusive superconducting/ferromagnetic hybrid structures with spin-active interfaces. Physical Review B.

[57] Aikebaier F, Silaev MA, Heikkilä TT (2018). Supercurrent-induced charge–spin conversion in spin-split superconductors. Physical Review B.

