# Supplementary material for: Controlling spin supercurrents via nonequilibrium spin injection
Source: Sci Rep. 2019 Sep 13;9:12731. doi: 10.1038/s41598-019-48945-0 (PMC6744513; doi:10.1038/s41598-019-48945-0)
Supplement: Supplementary file 1 — Supplementary Information [file 41598_2019_48945_MOESM1_ESM.pdf]

Supplemental information for "Controlling spin supercurrents via nonequilibrium spin injection"

Jabir Ali Ouassou,[1] Jason W. A. Robinson,[2] and Jacob Linder[1]

[1]*Center for Quantum Spintronics, Department of Physics, Norwegian University of Science and Technology, NO-7491 Trondheim, Norway*

[2]*Department of Materials Science and Metallurgy, University of Cambridge, 27 Charles Babbage Road, Cambridge CB3 0FS, United Kingdom*

Section I provides a self-contained derivation of the charge and spin transport equations. Specifically, we start with the definitions of particle densities from quantum field theory, and derive quasiparticle continuity equations. The results are used to derive quasiclassical results for the charge accumulation, spin accumulation, charge current, and spin current. Section II then uses these results to derive an analytical expression for the spin supercurrent in materials with spin accumulation. The result is used to explain the predictions in the main manuscript. Finally, in Section III, we discuss some technical details about the numerical model used for Figs. 2–3 in the main article.

I. CHARGE AND SPIN TRANSPORT

A. Quasiparticle accumulations

There are four relevant species of quasiparticles in the systems that we will consider: namely electrons and holes, which each have two distinct spin projections. These have the densities

$$n_{e\uparrow}(\boldsymbol{r},t) := \left\langle \Psi^\dagger_\uparrow(\boldsymbol{r},t)\,\Psi_\uparrow(\boldsymbol{r},t)\right\rangle, \tag{S1}$$

$$n_{e\downarrow}(\boldsymbol{r},t) := \left\langle \Psi^\dagger_\downarrow(\boldsymbol{r},t)\,\Psi_\downarrow(\boldsymbol{r},t)\right\rangle, \tag{S2}$$

$$n_{h\uparrow}(\boldsymbol{r},t) := \left\langle \Psi_\uparrow(\boldsymbol{r},t)\,\Psi^\dagger_\uparrow(\boldsymbol{r},t)\right\rangle, \tag{S3}$$

$$n_{h\downarrow}(\boldsymbol{r},t) := \left\langle \Psi_\downarrow(\boldsymbol{r},t)\,\Psi^\dagger_\downarrow(\boldsymbol{r},t)\right\rangle, \tag{S4}$$

where $\Psi^\dagger_\sigma$ and Ψ_σ are standard creation and annihilation operators. For comparison, the propagators are defined as [S1–S3]:

$$G^{\mathrm{R}}_{\sigma\sigma'}(\boldsymbol{r},t;\boldsymbol{r}',t') := -i\langle\{\Psi_\sigma(\boldsymbol{r},t),\,\Psi^\dagger_{\sigma'}(\boldsymbol{r}',t')\}\rangle\,\theta(t-t'), \tag{S5}$$

$$G^{\mathrm{A}}_{\sigma\sigma'}(\boldsymbol{r},t;\boldsymbol{r}',t') := +i\langle\{\Psi_\sigma(\boldsymbol{r},t),\,\Psi^\dagger_{\sigma'}(\boldsymbol{r}',t')\}\rangle\,\theta(t'-t), \tag{S6}$$

$$G^{\mathrm{K}}_{\sigma\sigma'}(\boldsymbol{r},t;\boldsymbol{r}',t') := -i\langle[\Psi_\sigma(\boldsymbol{r},t),\,\Psi^\dagger_{\sigma'}(\boldsymbol{r}',t')]\rangle, \tag{S7}$$

where the subscripts σ and σ' denote possible spin projections. Combining these definitions, we see that the quasiparticle densities are directly related to the equal-coordinate propagators:

$$n_{e\sigma} = \frac{i}{2}[G^{\mathrm{R}}_{\sigma\sigma} - G^{\mathrm{A}}_{\sigma\sigma} - G^{\mathrm{K}}_{\sigma\sigma}], \tag{S8}$$

$$n_{h\sigma} = \frac{i}{2}[G^{\mathrm{R}}_{\sigma\sigma} - G^{\mathrm{A}}_{\sigma\sigma} + G^{\mathrm{K}}_{\sigma\sigma}]. \tag{S9}$$

These expressions can be used to calculate the spin-resolved density of electrons and holes, respectively. Note that holes carry both opposite charge and opposite spin compared to electrons [S26]. The charge and spin accumulations are then found by multiplying each quasiparticle density with their respective charges or spins, and summing up their contributions:

$$\rho_{\mathrm{e}} := e\,\frac{1}{2}[+n_{e\uparrow} + n_{e\downarrow} - n_{h\uparrow} - n_{h\downarrow}], \tag{S10}$$

$$\rho_z := \frac{\hbar}{2}\frac{1}{2}[+n_{e\uparrow} - n_{e\downarrow} - n_{h\uparrow} + n_{h\downarrow}], \tag{S11}$$

where we use the convention that e is the *electron charge* ($e < 0$). The prefactors $1/2$ are required to prevent double-counting, and can be explained as follows. If we add one physical electron to the system, then the charge of the system increases by e. However, the number of electrons increases by one, and the number of holes decreases by one, meaning that the difference between electrons and holes increases by *two*. Thus, when the charge density ρ_{e} is described in terms of *both* electrons and holes, we need an extra factor $1/2$ to get the right physical charge. The same logic applies to the spin density ρ_z. We can rewrite the results in terms of the propagators above, and recognize the remaining sum as a trace over spins:

$$\rho_{\mathrm{e}} = -\frac{i}{2}e\;\mathrm{Tr}[\sigma_0 G^{\mathrm{K}}], \tag{S12}$$

$$\rho_z = -\frac{i}{2}\frac{\hbar}{2}\;\mathrm{Tr}[\sigma_3 G^{\mathrm{K}}]. \tag{S13}$$

There is nothing special about the spin-z axis, so it is straightforward to generalize this result to arbitrary spin-projections:

$$\rho_{\mathrm{e}} = -\frac{i}{2}e\;\mathrm{Tr}[\sigma_0 G^{\mathrm{K}}], \tag{S14}$$

$$\boldsymbol{\rho}_{\mathrm{s}} = -\frac{i}{2}\frac{\hbar}{2}\;\mathrm{Tr}[\boldsymbol{\sigma} G^{\mathrm{K}}], \tag{S15}$$

where $\boldsymbol{\sigma} = (\sigma_1, \sigma_2, \sigma_3)$ is the Pauli vector. From the definition of the Keldysh propagator above, we can also use the identity $\langle AB\rangle^* = \langle B^\dagger A^\dagger\rangle$ to show that $G^{\mathrm{K}*}_{\sigma\sigma} = -G^{\mathrm{K}}_{\sigma\sigma}$. This means that $G^{\mathrm{K}}_{\sigma\sigma}$ is imaginary, which makes $\rho_{\mathrm{e}}, \boldsymbol{\rho}_{\mathrm{s}} \sim iG^{\mathrm{K}}$ manifestly real. For later convenience, we will therefore write this out explicitly:

$$\rho_{\mathrm{e}} = -\frac{1}{2}e\;\mathrm{Re}\,\mathrm{Tr}[i\sigma_0 G^{\mathrm{K}}], \tag{S16}$$

$$\boldsymbol{\rho}_{\mathrm{s}} = -\frac{1}{2}\frac{\hbar}{2}\;\mathrm{Re}\,\mathrm{Tr}[i\boldsymbol{\sigma} G^{\mathrm{K}}]. \tag{S17}$$

B. Quasiparticle currents

Now that we know the charge and spin accumulations, the next step is to find the corresponding currents. To derive these, we go back to the quasiparticle densities defined in Eq. (S1):

$$n_{e\sigma}(\boldsymbol{r},t) := \left\langle \Psi^\dagger_\sigma(\boldsymbol{r},t)\,\Psi_\sigma(\boldsymbol{r},t)\right\rangle, \tag{S18}$$

$$n_{h\sigma}(\boldsymbol{r},t) := \left\langle \Psi_\sigma(\boldsymbol{r},t)\,\Psi^\dagger_\sigma(\boldsymbol{r},t)\right\rangle. \tag{S19}$$

To rigorously derive expressions for the charge and spin currents, we will use the definitions above to look for quasiparticle continuity equations on the form

$$\partial_t n_{\tau\sigma} + \nabla \cdot \boldsymbol{j}_{\tau\sigma} = q_{\tau\sigma}, \tag{S20}$$

where $\boldsymbol{j}_{\tau\sigma}$ is the particle- and spin-resolved current density we are interested in, while $q_{\tau\sigma}$ represents possible source terms.

We start by differentiating the densities with respect to time:

$$\partial_t n_{e\sigma} = \big\langle (\partial_t \Psi^\dagger_\sigma)\, \Psi_\sigma + \Psi^\dagger_\sigma\, (\partial_t \Psi_\sigma) \big\rangle, \tag{S21}$$

$$\partial_t n_{h\sigma} = \big\langle (\partial_t \Psi_\sigma)\, \Psi^\dagger_\sigma + \Psi_\sigma\, (\partial_t \Psi^\dagger_\sigma) \big\rangle. \tag{S22}$$

We can rewrite the above using the Heisenberg equation of motion for the field operators. Note that any contributions to the continuity equation arising from non-derivative terms in the Hamiltonian—such as a superconducting gap or an exchange field—can be incorporated into the source term q. Thus, for the purposes of deriving current equations, it is sufficient to consider only derivative terms. Whether or not the currents we derive are *conserved* currents can be checked at the end of the derivation, by substituting the Usadel equation into the final quasiclassical current equations [S4, S5]. If we for simplicity disregard gauge fields for now, the equations reduce to:

$$\partial_t \Psi_\sigma = +\frac{i}{2m} \nabla^2 \Psi_\sigma, \tag{S23}$$

$$\partial_t \Psi^\dagger_\sigma = -\frac{i}{2m} \nabla^2 \Psi^\dagger_\sigma. \tag{S24}$$

We then substitute these back into the equations for $\partial_t n_{\tau\sigma}$:

$$\partial_t n_{e\sigma} = -\frac{i}{2m} \big\langle (\nabla^2 \Psi^\dagger_\sigma)\, \Psi_\sigma - \Psi^\dagger_\sigma\, (\nabla^2 \Psi_\sigma) \big\rangle, \tag{S25}$$

$$\partial_t n_{h\sigma} = +\frac{i}{2m} \big\langle (\nabla^2 \Psi_\sigma)\, \Psi^\dagger_\sigma - \Psi_\sigma\, (\nabla^2 \Psi^\dagger_\sigma) \big\rangle. \tag{S26}$$

Thanks to cancellation of cross-terms, these can be factorized:

$$\partial_t n_{e\sigma} = -\frac{i}{2m} \nabla \cdot \big\langle (\nabla \Psi^\dagger_\sigma)\, \Psi_\sigma - \Psi^\dagger_\sigma\, (\nabla \Psi_\sigma) \big\rangle, \tag{S27}$$

$$\partial_t n_{h\sigma} = +\frac{i}{2m} \nabla \cdot \big\langle (\nabla \Psi_\sigma)\, \Psi^\dagger_\sigma - \Psi_\sigma\, (\nabla \Psi^\dagger_\sigma) \big\rangle. \tag{S28}$$

Comparing this to Eq. (S20), we conclude that:

$$j_{e\sigma} = -\frac{i}{2m} \big\langle (\nabla \Psi^\dagger_\sigma)\, \Psi_\sigma - \Psi^\dagger_\sigma\, (\nabla \Psi_\sigma) \big\rangle, \tag{S29}$$

$$j_{h\sigma} = +\frac{i}{2m} \big\langle (\nabla \Psi_\sigma)\, \Psi^\dagger_\sigma - \Psi_\sigma\, (\nabla \Psi^\dagger_\sigma) \big\rangle. \tag{S30}$$

As a mathematical trick, let us now use different coordinates $\Psi_\sigma = \Psi_\sigma(\boldsymbol{r}, t)$ and $\Psi^\dagger_\sigma = \Psi^\dagger_\sigma(\boldsymbol{r}', t')$ for the field operators, where we let $\boldsymbol{r}' \to \boldsymbol{r}$ and $t' \to t$ in the end. In this case, the differential operators acting on the field operators can be factored out of the expectation value without ambiguity:

$$j_{e\sigma} = -\frac{i}{2m} (\nabla' - \nabla) \big\langle \Psi^\dagger_\sigma(\boldsymbol{r}', t')\, \Psi_\sigma(\boldsymbol{r}, t) \big\rangle, \tag{S31}$$

$$j_{h\sigma} = -\frac{i}{2m} (\nabla' - \nabla) \big\langle \Psi_\sigma(\boldsymbol{r}, t)\, \Psi^\dagger_\sigma(\boldsymbol{r}', t') \big\rangle. \tag{S32}$$

We are now ready to define the charge and spin current densities. In correspondence with Eq. (S10), we define these as:

$$J_{\mathrm{e}} \coloneqq e\, \frac{1}{2} [+j_{e\uparrow} + j_{e\downarrow} - j_{h\uparrow} - j_{h\downarrow}], \tag{S33}$$

$$J_z \coloneqq \frac{\hbar}{2} \frac{1}{2} [+j_{e\uparrow} - j_{e\downarrow} - j_{h\uparrow} + j_{h\downarrow}]. \tag{S34}$$

Substituting Eq. (S31) into Eqs. (S33) and (S34), comparing the results to the Keldysh propagator in Eq. (S7), and recognizing the results as traces in spin space, we conclude:

$$J_{\mathrm{e}} = -e\, \frac{1}{4m} (\nabla' - \nabla)\ \mathrm{Tr}[\sigma_0 G^{\mathrm{K}}], \tag{S35}$$

$$J_z = -\frac{\hbar}{2} \frac{1}{4m} (\nabla' - \nabla)\ \mathrm{Tr}[\sigma_3 G^{\mathrm{K}}]. \tag{S36}$$

Generalizing to all spin projections, we obtain the final results:

$$J_{\mathrm{e}} = -e\, \frac{1}{4m} (\nabla' - \nabla)\ \mathrm{Tr}[\sigma_0 G^{\mathrm{K}}], \tag{S37}$$

$$\boldsymbol{J}_{\mathrm{s}} = -\frac{\hbar}{2} \frac{1}{4m} (\nabla' - \nabla)\ \mathrm{Tr}[\boldsymbol{\sigma} G^{\mathrm{K}}]. \tag{S38}$$

We wish to point out that these currents are manifestly real. From the definition of the Keldysh propagator, we see that:

$$(\nabla' - \nabla)[G^{\mathrm{K}*}(\boldsymbol{r}, t; \boldsymbol{r}', t')] = (\nabla' - \nabla)[-G^{\mathrm{K}}(\boldsymbol{r}', t'; \boldsymbol{r}, t)]. \tag{S39}$$

But which set of coordinates we chose to call $(\boldsymbol{r}, t)$ and $(\boldsymbol{r}', t')$ was arbitrary, and should not affect the physical results, since we are considering the limit $\boldsymbol{r}', t' \to \boldsymbol{r}, t$ anyway. This means that we can interchange the coordinates $(\boldsymbol{r}, t)$ and $(\boldsymbol{r}', t')$ on the right-hand side of the equation, *as long as we do this consistently for every factor simultaneously*. The coordinate interchange leads to a sign flip in $(\nabla' - \nabla)$ which cancels the minus sign inside the brackets, and makes the two sides of the equation equal. This lets us conclude that $(\nabla' - \nabla) G^{\mathrm{K}*} = (\nabla' - \nabla) G^{\mathrm{K}}$, which in turn implies that the charge and spin currents are real. For later convenience, we can therefore rewrite the above as

$$J_{\mathrm{e}} = -e\, \frac{1}{4m}\ \mathrm{Re}\,\mathrm{Tr}[\sigma_0 (\nabla' - \nabla)\, G^{\mathrm{K}}], \tag{S40}$$

$$\boldsymbol{J}_{\mathrm{s}} = -\frac{\hbar}{2} \frac{1}{4m}\ \mathrm{Re}\,\mathrm{Tr}[\boldsymbol{\sigma} (\nabla' - \nabla)\, G^{\mathrm{K}}]. \tag{S41}$$

C. Quasiclassical and diffusive limits

To derive equations we can use together with the Usadel equation, we now follow the standard prescription for taking the quasiclassical and diffusive limits [S1–S3, S6]. The net change to the Keldysh propagator and its derivative are then:

$$iG^{\mathrm{K}} \to \frac{1}{4} N_0 \int_{-\infty}^{+\infty} \mathrm{d}\epsilon\ \langle g^{\mathrm{K}} \rangle_{\mathrm{F}}, \tag{S42}$$

$$\frac{1}{2m} (\nabla' - \nabla) G^{\mathrm{K}} \to \frac{1}{4} N_0 \int_{-\infty}^{+\infty} \mathrm{d}\epsilon\ \langle \boldsymbol{v} g^{\mathrm{K}} \rangle_{\mathrm{F}}, \tag{S43}$$

where $\boldsymbol{v} := \boldsymbol{p}/m$ is interpreted as the quasiparticle velocity, N_0 is the density of states at the Fermi level, and $\langle\cdots\rangle_{\text{F}}$ refers to the average over the Fermi surface. From the derivation of the Usadel equation, we also know that in the diffusive limit the Fermi-surface averages can be written [S6, S7]

$$\langle\check{g}\rangle_{\text{F}} := \check{g}_s, \qquad \langle\boldsymbol{v}\check{g}\rangle_{\text{F}} \approx -D(\check{g}_s\tilde{\nabla}\check{g}_s), \tag{S44}$$

where $\tilde{\nabla}$ is a gauge-covariant derivative including the electromagnetic vector potential and spin-orbit interactions [S6, S7, S20], $\check{g}_s$ is the isotropic propagator, and D is the diffusion constant. We drop the subscripts on the isotropic propagators $\check{g}_s$, and substitute the above into the accumulations and currents:

$$\rho_{\text{e}} = -e\,\frac{1}{8}N_0\int_{-\infty}^{+\infty} \mathrm{d}\epsilon\ \operatorname{Re}\operatorname{Tr}[\sigma_0 g^{\text{K}}], \tag{S45}$$

$$\boldsymbol{\rho}_{\text{s}} = -\frac{\hbar}{2}\frac{1}{8}N_0\int_{-\infty}^{+\infty} \mathrm{d}\epsilon\ \operatorname{Re}\operatorname{Tr}[\boldsymbol{\sigma} g^{\text{K}}], \tag{S46}$$

$$J_{\text{e}} = +e\,\frac{1}{8}N_0\int_{-\infty}^{+\infty} \mathrm{d}\epsilon\ \operatorname{Re}\operatorname{Tr}[\sigma_0 \boldsymbol{I}^{\text{K}}], \tag{S47}$$

$$\boldsymbol{J}_{\text{s}} = +\frac{\hbar}{2}\frac{1}{8}N_0\int_{-\infty}^{+\infty} \mathrm{d}\epsilon\ \operatorname{Re}\operatorname{Tr}[\boldsymbol{\sigma} \boldsymbol{I}^{\text{K}}], \tag{S48}$$

where we have reintroduced the matrix current $\check{\boldsymbol{I}} := D(\check{g}\tilde{\nabla}\check{g})$. Note that these equations only depend on the "electronic" part of the propagators in Nambu space, which in reality contains information about both the electrons and holes in the system.

All these results can be written as integrals over only positive energies using the symmetries of the Nambu-space matrices

$$\hat{\sigma}_n\hat{g}^{\text{K}}(\epsilon) = \begin{pmatrix} +\sigma_n g^{\text{K}}(+\epsilon) & +\sigma_n f^{\text{K}}(+\epsilon) \\ +\sigma_n^* f^{\text{K}*}(-\epsilon) & +\sigma_n^* g^{\text{K}*}(-\epsilon) \end{pmatrix}, \tag{S49}$$

$$\hat{\sigma}_n\hat{\boldsymbol{I}}^{\text{K}}(\epsilon) = \begin{pmatrix} +\sigma_n \boldsymbol{I}^{\text{K}}(+\epsilon) & +\sigma_n \boldsymbol{J}^{\text{K}}(+\epsilon) \\ -\sigma_n^* \boldsymbol{J}^{\text{K}*}(-\epsilon) & -\sigma_n^* \boldsymbol{I}^{\text{K}*}(-\epsilon) \end{pmatrix}. \tag{S50}$$

In other words, the negative-energy contributions can be recast in terms of the lower-right blocks; and since take the real part of the results, the complex conjugations are irrelevant. The remaining structure can be recognized as a trace over Nambu space, yielding the final quasiclassical transport equations

$$\rho_{\text{e}} = -e\,\frac{1}{8}N_0\int_0^{\infty} \mathrm{d}\epsilon\ \operatorname{Re}\operatorname{Tr}[\hat{\tau}_0\hat{\sigma}_0\hat{g}^{\text{K}}], \tag{S51}$$

$$\boldsymbol{\rho}_{\text{s}} = -\frac{\hbar}{2}\frac{1}{8}N_0\int_0^{\infty} \mathrm{d}\epsilon\ \operatorname{Re}\operatorname{Tr}[\hat{\tau}_0\hat{\boldsymbol{\sigma}}\hat{g}^{\text{K}}], \tag{S52}$$

$$J_{\text{e}} = +e\,\frac{1}{8}N_0\int_0^{\infty} \mathrm{d}\epsilon\ \operatorname{Re}\operatorname{Tr}[\hat{\tau}_3\hat{\sigma}_0\hat{\boldsymbol{I}}^{\text{K}}], \tag{S53}$$

$$\boldsymbol{J}_{\text{s}} = +\frac{\hbar}{2}\frac{1}{8}N_0\int_0^{\infty} \mathrm{d}\epsilon\ \operatorname{Re}\operatorname{Tr}[\hat{\tau}_3\hat{\boldsymbol{\sigma}}\hat{\boldsymbol{I}}^{\text{K}}]. \tag{S54}$$

Note that $\hat{\boldsymbol{\sigma}}\hat{\boldsymbol{I}}^{\text{K}}$ should be interpreted as an outer product between two vectors, which results in a rank-2 tensor. This is because a general description of spin transport requires both a direction of transport $\sim\hat{\boldsymbol{I}}^{\text{K}}$ and a spin orientation $\sim\hat{\boldsymbol{\sigma}}$.

D. Higher-order gauge contributions

The equations of motion for the field operators also include first-order derivative terms in systems with electromagnetic [S6, S9] or spin-orbit [S7, S10, S11, S20] gauge fields. If we ignore all other terms in the Hamiltonian, these derivative terms give the following Heisenberg equations:

$$\partial_t\Psi_\sigma = \frac{1}{m}\boldsymbol{A}_{\sigma\sigma'}\cdot(\nabla\Psi_{\sigma'}), \tag{S55}$$

$$\partial_t\Psi_\sigma^\dagger = \frac{1}{m}(\nabla\Psi_{\sigma'}^\dagger)\cdot\boldsymbol{A}_{\sigma'\sigma}, \tag{S56}$$

where we implicitly sum over the spin index σ'. Going through the same kind of derivations as without the gauge fields, we find that we basically just have to make the following replacement in the results right before taking the quasiclassical limit:

$$\boldsymbol{p}G^{\text{K}} \to \frac{1}{2}\{G^{\text{K}}, \boldsymbol{p}-\boldsymbol{A}\}. \tag{S57}$$

Note that the gauge fields also affects charge and spin transport in a different way, since they also appear as covariant derivatives $\tilde{\nabla}(\,\cdot\,) = \nabla(\,\cdot\,) - i[\boldsymbol{A},\,\cdot\,]$ in the matrix current $\check{I} = D\check{g}\tilde{\nabla}\check{g}$.

II. NONEQUILIBRIUM SUPERCURRENTS

A. Supercurrents vs. resistive currents

As shown in previous sections, the total spin current $\boldsymbol{J}_{\text{s}}$ can in the quasiclassical limit be calculated as an energy integral,

$$\boldsymbol{J}_{\text{s}} = \frac{\hbar}{2}N_0\int_0^{\infty}\mathrm{d}\epsilon\ \boldsymbol{j}_{\text{s}}, \tag{S58}$$

where the spectral spin current $\boldsymbol{j}_{\text{s}} := \operatorname{Re}\operatorname{Tr}[\hat{\tau}_3\hat{\boldsymbol{\sigma}}\hat{\boldsymbol{I}}^{\text{K}}]/8$ and the matrix current $\check{\boldsymbol{I}} := D\check{g}\nabla\check{g}$. If we substitute the parametrization $\hat{g}^{\text{K}} = \hat{g}^{\text{R}}\hat{h} - \hat{h}\hat{g}^{\text{A}}$ into the definition of the matrix current, we find that its Keldysh component can be expanded as

$$\begin{aligned}\hat{\boldsymbol{I}}^{\text{K}} &= D[(\hat{g}^{\text{R}}\nabla\hat{g}^{\text{R}})\hat{h} - \hat{h}(\hat{g}^{\text{A}}\nabla\hat{g}^{\text{A}})] \\ &+ D[(\nabla\hat{h}) - \hat{g}^{\text{R}}(\nabla\hat{h})\hat{g}^{\text{A}}].\end{aligned} \tag{S59}$$

The terms on the first line may be finite even for a homogeneous distribution function $\hat{h}$, and produces spin currents even in equilibrium. Furthermore, they are sensitive to the phase-winding of the superconducting condensate via $\hat{g}^{\text{R}}\nabla\hat{g}^{\text{R}}$ and $\hat{g}^{\text{A}}\nabla\hat{g}^{\text{A}}$. We therefore identify this as a supercurrent contribution. The terms on the second line, however, are proportional to $\nabla\hat{h}$. This current contribution both requires an inhomogeneous distribution function, and is insensitive to the phase-winding of the superconducting condensate, and has to be a resistive current.

In this work, we are primarily interested in generating a spin supercurrent from a nonequilibrium spin accumulation. We therefore limit our attention to systems with a position-independent distribution function $\hat{h}$ that has an excited spin mode. Since we assume $\nabla\hat{h} = 0$, the second line of Eq. (S59) disappears, and only the supercurrent contribution remains:

$$\boldsymbol{j}_\mathrm{s} = \frac{1}{8}\,\mathrm{Re}\,\mathrm{Tr}\left[\hat{h}\hat{\tau}_3\hat{\boldsymbol{\sigma}}(\hat{g}^\mathrm{R}\nabla\hat{g}^\mathrm{R}) - \hat{\tau}_3\hat{\boldsymbol{\sigma}}\hat{h}(\hat{g}^{\mathrm{R}\dagger}\nabla\hat{g}^{\mathrm{R}\dagger})\right]. \tag{S60}$$

As for the distribution function, it can be parametrized as

$$\hat{h} = h_0\hat{\sigma}_0\hat{\tau}_0 + \boldsymbol{h}_\mathrm{s}\cdot\hat{\boldsymbol{\sigma}}\hat{\tau}_3, \tag{S61}$$

where $\boldsymbol{h}_\mathrm{s}$ points along the net quantization axis of the accumulated spins, and the magnitudes of the modes above are

$$h_0(\epsilon) = \tfrac{1}{2}\tanh[(\epsilon + eV_\mathrm{s})/2T] + \tfrac{1}{2}\tanh[(\epsilon - eV_\mathrm{s})/2T], \tag{S62}$$
$$h_\mathrm{s}(\epsilon) = \tfrac{1}{2}\tanh[(\epsilon + eV_\mathrm{s})/2T] - \tfrac{1}{2}\tanh[(\epsilon - eV_\mathrm{s})/2T]. \tag{S63}$$

Note that the energy mode h_0 and spin mode h_s are odd and even functions of energy, respectively. We have parametrized the spin mode in terms of a spin voltage $V_\mathrm{s} := (V_\uparrow - V_\downarrow)/2$, where V_σ are the effective potentials experienced by spin-σ quasiparticles [S12–S14]. The spin mode $\boldsymbol{h}_\mathrm{s}$ is related to the spin accumulation in Eq. (S52) by an energy integral $\boldsymbol{\rho}_\mathrm{s} \sim \int \mathrm{d}\epsilon\;\, N(\epsilon)\,\boldsymbol{h}_\mathrm{s}(\epsilon)$, where $N(\epsilon)$ is the density of states [S13].

B. Expansion in Pauli matrices

Once we substitute Eq. (S61) into Eq. (S60), there are a few subtleties to be careful about. To handle these, without yet introducing all the details of the singlet/triplet-decomposition, we first expand $\hat{g}^\mathrm{R}\nabla\hat{g}^\mathrm{R}$ directly in terms of Pauli matrices:

$$\hat{g}^\mathrm{R}\nabla\hat{g}^\mathrm{R} := \boldsymbol{\alpha}\cdot\hat{\boldsymbol{\sigma}}\hat{\tau}_3 + \boldsymbol{\beta}\cdot\hat{\boldsymbol{\sigma}}\hat{\tau}_0 + \gamma\,\hat{\sigma}_0\hat{\tau}_3 + \delta\,\hat{\sigma}_0\hat{\tau}_0 + \hat{\epsilon}. \tag{S64}$$

The first four terms parametrizes a general block-diagonal matrix, while the last term $\hat{\epsilon}$ represents off-block-diagonal parts. Since the distribution $\hat{h}$ can always be chosen to be block-diagonal, $\hat{\epsilon}$ does not contribute to the trace in Eq. (S60). The other coefficients are found by taking appropriate traces:

$$\begin{aligned} \boldsymbol{\alpha} &= \frac{1}{4}\,\mathrm{Tr}[\hat{\boldsymbol{\sigma}}\hat{\tau}_3\hat{g}^\mathrm{R}\nabla\hat{g}^\mathrm{R}], & \boldsymbol{\beta} &= \frac{1}{4}\,\mathrm{Tr}[\hat{\boldsymbol{\sigma}}\hat{\tau}_0\hat{g}^\mathrm{R}\nabla\hat{g}^\mathrm{R}], \\ \gamma &= \frac{1}{4}\,\mathrm{Tr}[\hat{\sigma}_0\hat{\tau}_3\hat{g}^\mathrm{R}\nabla\hat{g}^\mathrm{R}], & \delta &= \frac{1}{4}\,\mathrm{Tr}[\hat{\sigma}_0\hat{\tau}_0\hat{g}^\mathrm{R}\nabla\hat{g}^\mathrm{R}]. \end{aligned} \tag{S65}$$

We parametrize $\hat{g}^{\mathrm{R}\dagger}\nabla\hat{g}^{\mathrm{R}\dagger}$ using coefficients $\underline{\boldsymbol{\alpha}}, \underline{\boldsymbol{\beta}}, \underline{\gamma}, \underline{\delta}$ that are defined in the same manner as above.

We will now argue that the parameter δ is identically zero. By differentiating the normalization condition $(\hat{g}^\mathrm{R})^2 = 1$, one can show that the retarded propagator anticommutes with its gradient, $\{\hat{g}^\mathrm{R}, \nabla\hat{g}^\mathrm{R}\} = 0$. This identity can be rewritten

$$\hat{g}^\mathrm{R}(\nabla\hat{g}^\mathrm{R}) = -(\nabla\hat{g}^\mathrm{R})\hat{g}^\mathrm{R}. \tag{S66}$$

Let us now trace both sides of the equation, and use the cyclic rule $\mathrm{Tr}[\hat{A}\hat{B}] = \mathrm{Tr}[\hat{B}\hat{A}]$ on the right-hand side,

$$\mathrm{Tr}[\hat{g}^\mathrm{R}(\nabla\hat{g}^\mathrm{R})] = -\,\mathrm{Tr}[\hat{g}^\mathrm{R}(\nabla\hat{g}^\mathrm{R})]. \tag{S67}$$

Since $\hat{\sigma}_0\hat{\tau}_0$ is an identity matrix, we see from Eq. (S65) that:

$$\delta = -\delta. \tag{S68}$$

In other words, $\delta = 0$ is always satisfied, as any other conclusion would violate the normalization condition $(\hat{g}^\mathrm{R})^2 = 1$.

Next, to clarify another subtlety, we need to derive some trace identities. By explicitly writing out the matrix products and using $\hat{\boldsymbol{\sigma}} = \mathrm{diag}(\boldsymbol{\sigma}, \boldsymbol{\sigma}^*)$, one can show that

$$\begin{aligned} \mathrm{Tr}[(\boldsymbol{a}\cdot\hat{\boldsymbol{\sigma}})(\boldsymbol{b}\cdot\hat{\boldsymbol{\sigma}})\hat{\boldsymbol{\sigma}}\hat{\tau}_0] &= \mathrm{Tr}[(\boldsymbol{a}\cdot\boldsymbol{\sigma}\;\,)(\boldsymbol{b}\cdot\boldsymbol{\sigma}\;\,)\boldsymbol{\sigma}\;\,] \\ &\quad + \mathrm{Tr}[(\boldsymbol{a}\cdot\boldsymbol{\sigma}^*)(\boldsymbol{b}\cdot\boldsymbol{\sigma}^*)\boldsymbol{\sigma}^*], \end{aligned} \tag{S69}$$

$$\begin{aligned} \mathrm{Tr}[(\boldsymbol{a}\cdot\hat{\boldsymbol{\sigma}})(\boldsymbol{b}\cdot\hat{\boldsymbol{\sigma}})\hat{\boldsymbol{\sigma}}\hat{\tau}_3] &= \mathrm{Tr}[(\boldsymbol{a}\cdot\boldsymbol{\sigma}\;\,)(\boldsymbol{b}\cdot\boldsymbol{\sigma}\;\,)\boldsymbol{\sigma}\;\,] \\ &\quad - \mathrm{Tr}[(\boldsymbol{a}\cdot\boldsymbol{\sigma}^*)(\boldsymbol{b}\cdot\boldsymbol{\sigma}^*)\boldsymbol{\sigma}^*]. \end{aligned} \tag{S70}$$

Products of spin matrices in general satisfy $(\boldsymbol{a}\cdot\boldsymbol{\sigma})(\boldsymbol{b}\cdot\boldsymbol{\sigma}) = (\boldsymbol{a}\cdot\boldsymbol{b}) + i(\boldsymbol{a}\times\boldsymbol{b})\cdot\boldsymbol{\sigma}$; multiplying by $\boldsymbol{\sigma}$ and taking the trace, we find the associated trace rule $\mathrm{Tr}[(\boldsymbol{a}\cdot\boldsymbol{\sigma})(\boldsymbol{b}\cdot\boldsymbol{\sigma})\boldsymbol{\sigma}] = +2i(\boldsymbol{a}\times\boldsymbol{b})$. However, if we complex-conjugate before taking the trace, we uncover another identity $\mathrm{Tr}[(\boldsymbol{a}\cdot\boldsymbol{\sigma}^*)(\boldsymbol{b}\cdot\boldsymbol{\sigma}^*)\boldsymbol{\sigma}^*] = -2i(\boldsymbol{a}\times\boldsymbol{b})$. A geometric motivation for the sign difference is that if the basis $\boldsymbol{\sigma} = (\sigma_1, \sigma_2, \sigma_3)$ defines a right-handed coordinate system, then $\boldsymbol{\sigma}^* = (\sigma_1, -\sigma_2, \sigma_3)$ has to define a left-handed one—and this inverts the right-hand rule that cross-products usually satisfy. With the aid of the results above, we see that

$$\mathrm{Tr}[(\boldsymbol{a}\cdot\hat{\boldsymbol{\sigma}})(\boldsymbol{b}\cdot\hat{\boldsymbol{\sigma}})\hat{\boldsymbol{\sigma}}\hat{\tau}_0] = 0, \tag{S71}$$
$$\mathrm{Tr}[(\boldsymbol{a}\cdot\hat{\boldsymbol{\sigma}})(\boldsymbol{b}\cdot\hat{\boldsymbol{\sigma}})\hat{\boldsymbol{\sigma}}\hat{\tau}_3] = 4i(\boldsymbol{a}\times\boldsymbol{b}). \tag{S72}$$

This is the subtle trap alluded to above: due to the way we define $\hat{\boldsymbol{\sigma}} = \mathrm{diag}(\boldsymbol{\sigma}, \boldsymbol{\sigma}^*)$, the generalization of the Pauli cross-product identity to matrices in Nambu space requires an extra factor $\hat{\tau}_3$ in the trace to produce a nonzero result.

We now substitute Eqs. (S61) and (S64) into Eq. (S60). With the identities above, we see that the only contributions are:

$$\boldsymbol{j}_\mathrm{s} = \tfrac{1}{2}h_0\,\mathrm{Re}\left[\boldsymbol{\alpha} - \underline{\boldsymbol{\alpha}}\right] + \tfrac{1}{2}\boldsymbol{h}_\mathrm{s}\times\mathrm{Im}\left[\boldsymbol{\alpha} + \underline{\boldsymbol{\alpha}}\right]. \tag{S73}$$

By multiplying Eq. (S66) by appropriate Pauli matrices, taking traces, and using $\mathrm{Tr}[\hat{A}^\dagger] = \mathrm{Tr}[\hat{A}]^*$, one can show that $\underline{\boldsymbol{\alpha}} = -\boldsymbol{\alpha}^*$. This makes $\boldsymbol{\alpha}-\underline{\boldsymbol{\alpha}}$ real and $\boldsymbol{\alpha}+\underline{\boldsymbol{\alpha}}$ imaginary, so both contributions are compatible with the normalization condition. We could also use this information to eliminate the underlined coefficients, but this would make it harder to see how mixed singlet/triplet-terms cancel later in the derivation. Interestingly, all spin supercurrent contributions depend on the same coefficient $\boldsymbol{\alpha}$, and do not couple to the other traces of $\hat{g}^\mathrm{R}\nabla\hat{g}^\mathrm{R}$.

The physically observable spin supercurrent is found by integrating the spectral current over all positive and negative energies. We also know that h_0 and $\boldsymbol{h}_\mathrm{s}$ are odd and even functions of energy, respectively. We can therefore let $\underline{\boldsymbol{\alpha}}(+\epsilon) \to \mp\underline{\boldsymbol{\alpha}}(-\epsilon) = \mp\underline{\tilde{\boldsymbol{\alpha}}}^*(+\epsilon)$ in the spectral current without changing the total spin supercurrent:

$$\boldsymbol{j}_\mathrm{s} = \tfrac{1}{2}h_0\,\mathrm{Re}\left[\boldsymbol{\alpha} + \underline{\tilde{\boldsymbol{\alpha}}}^*\right] + \tfrac{1}{2}\boldsymbol{h}_\mathrm{s}\times\mathrm{Im}\left[\boldsymbol{\alpha} + \underline{\tilde{\boldsymbol{\alpha}}}^*\right]. \tag{S74}$$

This form of the result will be useful later, as it makes it clearer which parts of the non-underlined and underlined coefficients cancel for symmetry reasons. Conveniently, this also makes the h_0 and $\boldsymbol{h}_\mathrm{s}$ contributions take very similar forms.

C. Expansion in singlets and triplets

We now proceed with an expansion of the propagators in terms of physically meaningful components. Following the same kind of parametrization as Ref. S15, we can write

$$\hat{g}^{\mathrm{R}} = \begin{pmatrix} (g_s + \boldsymbol{g}_t \cdot \boldsymbol{\sigma}) & (f_s + \boldsymbol{f}_t \cdot \boldsymbol{\sigma}) i\sigma_2 \\ -i\sigma_2(\tilde{f}_s - \tilde{\boldsymbol{f}}_t \cdot \boldsymbol{\sigma}) & -\sigma_2(\tilde{g}_s - \tilde{\boldsymbol{g}}_t \cdot \boldsymbol{\sigma})\sigma_2 \end{pmatrix}. \tag{S75}$$

Here, f_s represents the spin-singlet pair amplitude, while $\boldsymbol{f}_t$ is the spin-triplet amplitude. On the other hand, we can interpret g_s and $\boldsymbol{g}_t$ as the spin-independent and spin-dependent parts of the density of states, respectively [S15]. In our notation, this means that the density of states for particles with spin-projection $\boldsymbol{p}$ is given by $N = N_0\,\mathrm{Re}[g_s + \boldsymbol{g}_t \cdot \boldsymbol{p}]$. In equilibrium, the spin accumulation is found by integrating $h_0\boldsymbol{g}_t$ over energies, giving another interpretation of $\boldsymbol{g}_t$. Outside of equilibrium, we of course get another kind of spin accumulation due to a nonzero spin mode $\boldsymbol{h}_{\mathrm{s}}$, which we are interested in here.

Using Eq. (S75) and the identity $\sigma_2\boldsymbol{\sigma}\sigma_2 = -\boldsymbol{\sigma}^*$, we find that the diagonal components of $\hat{g}^{\mathrm{R}}\nabla\hat{g}^{\mathrm{R}}$ in Nambu space are

$$\begin{aligned} \big[\hat{g}^{\mathrm{R}}\nabla\hat{g}^{\mathrm{R}}\big]_{1,1} &= (g_s + \boldsymbol{g}_t \cdot \boldsymbol{\sigma})\nabla(g_s + \boldsymbol{g}_t \cdot \boldsymbol{\sigma}) \\ &\quad + (f_s + \boldsymbol{f}_t \cdot \boldsymbol{\sigma})\nabla(\tilde{f}_s - \tilde{\boldsymbol{f}}_t \cdot \boldsymbol{\sigma}), \\ \big[\hat{g}^{\mathrm{R}}\nabla\hat{g}^{\mathrm{R}}\big]_{2,2} &= (\tilde{g}_s + \tilde{\boldsymbol{g}}_t \cdot \boldsymbol{\sigma}^*)\nabla(\tilde{g}_s + \tilde{\boldsymbol{g}}_t \cdot \boldsymbol{\sigma}^*) \\ &\quad + (\tilde{f}_s + \tilde{\boldsymbol{f}}_t \cdot \boldsymbol{\sigma}^*)\nabla(f_s - \boldsymbol{f}_t \cdot \boldsymbol{\sigma}^*), \end{aligned} \tag{S76}$$

where the subscripts $[\cdots]_{i,j}$ are matrix indices in Nambu space. Using the identity $(\boldsymbol{a}\cdot\boldsymbol{\sigma})(\boldsymbol{b}\cdot\boldsymbol{\sigma}) = (\boldsymbol{a}\cdot\boldsymbol{b}) + i(\boldsymbol{a}\times\boldsymbol{b})\cdot\boldsymbol{\sigma}$ and its conjugate $(\boldsymbol{a}\cdot\boldsymbol{\sigma}^*)(\boldsymbol{b}\cdot\boldsymbol{\sigma}^*) = (\boldsymbol{a}\cdot\boldsymbol{b}) - i(\boldsymbol{a}\times\boldsymbol{b})\cdot\boldsymbol{\sigma}^*$, we can sort the above into spin-independent and spin-dependent terms,

$$\begin{aligned} \big[\hat{g}^{\mathrm{R}}\nabla\hat{g}^{\mathrm{R}}\big]_{1,1} &= \big(g_s\nabla g_s + \boldsymbol{g}_t\nabla\boldsymbol{g}_t + f_s\nabla\tilde{f}_s - \boldsymbol{f}_t\nabla\tilde{\boldsymbol{f}}_t\big) \\ &\quad + \big(g_s\nabla\boldsymbol{g}_t + \boldsymbol{g}_t\nabla g_s + \boldsymbol{f}_t\nabla\tilde{f}_s - f_s\nabla\tilde{\boldsymbol{f}}_t\big)\boldsymbol{\sigma} \\ &\quad + \big(\boldsymbol{g}_t\times\nabla\boldsymbol{g}_t - \boldsymbol{f}_t\times\nabla\tilde{\boldsymbol{f}}_t\big)i\boldsymbol{\sigma}, \\ \big[\hat{g}^{\mathrm{R}}\nabla\hat{g}^{\mathrm{R}}\big]_{2,2} &= \big(\tilde{g}_s\nabla\tilde{g}_s + \tilde{\boldsymbol{g}}_t\nabla\tilde{\boldsymbol{g}}_t + \tilde{f}_s\nabla f_s - \tilde{\boldsymbol{f}}_t\nabla\boldsymbol{f}_t\big) \\ &\quad + \big(\tilde{g}_s\nabla\tilde{\boldsymbol{g}}_t + \tilde{\boldsymbol{g}}_t\nabla\tilde{g}_s + \tilde{\boldsymbol{f}}_t\nabla f_s - \tilde{f}_s\nabla\boldsymbol{f}_t\big)\boldsymbol{\sigma}^* \\ &\quad - \big(\tilde{\boldsymbol{g}}_t\times\nabla\tilde{\boldsymbol{g}}_t - \tilde{\boldsymbol{f}}_t\times\nabla\boldsymbol{f}_t\big)i\boldsymbol{\sigma}^*. \end{aligned} \tag{S77}$$

Since we define $\hat{\boldsymbol{\sigma}} = \mathrm{diag}(\boldsymbol{\sigma}, \boldsymbol{\sigma}^*)$, Eq. (S65) tells us that the coefficient $\boldsymbol{\alpha}$ that we require can be expressed as

$$\boldsymbol{\alpha} = \frac{1}{4}\,\mathrm{Tr}\left\{\boldsymbol{\sigma}[\hat{g}^{\mathrm{R}}\nabla\hat{g}^{\mathrm{R}}]_{1,1} - \boldsymbol{\sigma}^*[\hat{g}^{\mathrm{R}}\nabla\hat{g}^{\mathrm{R}}]_{2,2}\right\}. \tag{S78}$$

Together with the expansion of $\hat{g}^{\mathrm{R}}\nabla\hat{g}^{\mathrm{R}}$ above, and standard trace identities for Pauli matrices, we then obtain

$$\begin{aligned} 2\boldsymbol{\alpha} &= g_s\nabla\boldsymbol{g}_t + \boldsymbol{g}_t\nabla g_s - \tilde{g}_s\nabla\tilde{\boldsymbol{g}}_t - \tilde{\boldsymbol{g}}_t\nabla\tilde{g}_s \\ &\quad + \boldsymbol{f}_t\nabla\tilde{f}_s - f_s\nabla\tilde{\boldsymbol{f}}_t - \tilde{\boldsymbol{f}}_t\nabla f_s + \tilde{f}_s\nabla\boldsymbol{f}_t \\ &\quad + i\boldsymbol{g}_t\times\nabla\boldsymbol{g}_t + i\tilde{\boldsymbol{g}}_t\times\nabla\tilde{\boldsymbol{g}}_t \\ &\quad - i\boldsymbol{f}_t\times\nabla\tilde{\boldsymbol{f}}_t - i\tilde{\boldsymbol{f}}_t\times\nabla\boldsymbol{f}_t. \end{aligned} \tag{S79}$$

Let us now calculate the corresponding coefficient $\underline{\boldsymbol{\alpha}}$ from the matrix $\hat{g}^{\mathrm{R}\dagger}\nabla\hat{g}^{\mathrm{R}\dagger}$. Taking the complex-transpose of Eq. (S75),

$$\hat{g}^{\mathrm{R}\dagger} = \begin{pmatrix} (g_s^* + \boldsymbol{g}_t^* \cdot \boldsymbol{\sigma}) & (\tilde{f}_s^* - \tilde{\boldsymbol{f}}_t^* \cdot \boldsymbol{\sigma}) i\sigma_2 \\ -i\sigma_2(f_s^* + \boldsymbol{f}_t^* \cdot \boldsymbol{\sigma}) & -\sigma_2(\tilde{g}_s^* - \tilde{\boldsymbol{g}}_t^* \cdot \boldsymbol{\sigma})\sigma_2 \end{pmatrix}, \tag{S80}$$

we see that $\hat{g}^{\mathrm{R}\dagger}$ changed as follows compared to $\hat{g}^{\mathrm{R}}$:

$$g_s \to +g_s^*, \quad \boldsymbol{g}_t \to +\boldsymbol{g}_t^*, \quad f_s \to +\tilde{f}_s^*, \quad \boldsymbol{f}_t \to -\tilde{\boldsymbol{f}}_t^*. \tag{S81}$$

Other than these transformations, the parametrization is clearly identical, and the derivation of $\underline{\boldsymbol{\alpha}}$ becomes identical as well. If we in the end results also choose to let $\epsilon \to -\epsilon$, corresponding to a combination of complex-conjugation and tilde-conjugation, the net transformation rules become

$$g_s \to +\tilde{g}_s, \quad \boldsymbol{g}_t \to +\tilde{\boldsymbol{g}}_t, \quad f_s \to +f_s, \quad \boldsymbol{f}_t \to -\boldsymbol{f}_t. \tag{S82}$$

We can therefore simply perform the changes above to Eq. (S79) to get the corresponding equations for $\underline{\tilde{\boldsymbol{\alpha}}}^*$:

$$\begin{aligned} 2\underline{\tilde{\boldsymbol{\alpha}}}^* &= \tilde{g}_s\nabla\tilde{\boldsymbol{g}}_t + \tilde{\boldsymbol{g}}_t\nabla\tilde{g}_s - g_s\nabla\boldsymbol{g}_t - \boldsymbol{g}_t\nabla g_s \\ &\quad - \boldsymbol{f}_t\nabla\tilde{f}_s + f_s\nabla\tilde{\boldsymbol{f}}_t + \tilde{\boldsymbol{f}}_t\nabla f_s - \tilde{f}_s\nabla\boldsymbol{f}_t \\ &\quad + i\tilde{\boldsymbol{g}}_t\times\nabla\tilde{\boldsymbol{g}}_t + i\boldsymbol{g}_t\times\nabla\boldsymbol{g}_t \\ &\quad - i\boldsymbol{f}_t\times\nabla\tilde{\boldsymbol{f}}_t - i\tilde{\boldsymbol{f}}_t\times\nabla\boldsymbol{f}_t. \end{aligned} \tag{S83}$$

We are now ready to calculate the spectral spin supercurrent in terms of the singlet/triplet-decomposition. Adding up Eqs. (S79) and (S83), we see that all mixed singlet/triplet terms drop out, and we are left with only the cross-product terms:

$$\boldsymbol{\alpha} + \underline{\tilde{\boldsymbol{\alpha}}}^* = +i\boldsymbol{g}_t\times\nabla\boldsymbol{g}_t + i\tilde{\boldsymbol{g}}_t\times\nabla\tilde{\boldsymbol{g}}_t - i\tilde{\boldsymbol{f}}_t\times\nabla\boldsymbol{f}_t - i\boldsymbol{f}_t\times\nabla\tilde{\boldsymbol{f}}_t. \tag{S84}$$

Substituting this into Eq. (S74), we immediately see that:

$$\begin{aligned} \boldsymbol{j}_{\mathrm{s}} &= -\tfrac{1}{2}h_0\,\mathrm{Im}\left[\boldsymbol{g}_t\times\nabla\boldsymbol{g}_t + \tilde{\boldsymbol{g}}_t\times\nabla\tilde{\boldsymbol{g}}_t - \boldsymbol{f}_t\times\nabla\tilde{\boldsymbol{f}}_t - \tilde{\boldsymbol{f}}_t\times\nabla\boldsymbol{f}_t\right] \\ &\quad + \tfrac{1}{2}\boldsymbol{h}_{\mathrm{s}}\times\mathrm{Re}\left[\boldsymbol{g}_t\times\nabla\boldsymbol{g}_t + \tilde{\boldsymbol{g}}_t\times\nabla\tilde{\boldsymbol{g}}_t - \boldsymbol{f}_t\times\nabla\tilde{\boldsymbol{f}}_t - \tilde{\boldsymbol{f}}_t\times\nabla\boldsymbol{f}_t\right]. \end{aligned} \tag{S85}$$

Since h_0 and $\boldsymbol{h}_{\mathrm{s}}$ are odd and even functions of energy, and the observable spin current is the energy integral of the above, we can let $h_0\,\mathrm{Im}[\tilde{A}] \to h_0\,\mathrm{Im}[A]$ and $\boldsymbol{h}_{\mathrm{s}}\,\mathrm{Re}[\tilde{A}] \to \boldsymbol{h}_{\mathrm{s}}\,\mathrm{Re}[A]$ without changing any results. Applied to the above, we can summarize our results in the tidy and compact form

$$\begin{aligned} \boldsymbol{j}_{\mathrm{s}} &= -\,\mathrm{Im}\big[\boldsymbol{g}_t\times\nabla\boldsymbol{g}_t - \boldsymbol{f}_t\times\nabla\tilde{\boldsymbol{f}}_t\big]\cdot h_0 \\ &\quad - \mathrm{Re}\big[\boldsymbol{g}_t\times\nabla\boldsymbol{g}_t - \boldsymbol{f}_t\times\nabla\tilde{\boldsymbol{f}}_t\big]\times\boldsymbol{h}_{\mathrm{s}}. \end{aligned} \tag{S86}$$

We have shown earlier in the derivation that both contributions are compatible with the normalization condition. The fact that they did not cancel during the last simplification above, shows that both contributions are compatible with the energy symmetries of h_0 and $\boldsymbol{h}_{\mathrm{s}}$. Finally, we know that the contents of the brackets $\boldsymbol{g}_t\times\nabla\boldsymbol{g}_t - \boldsymbol{f}_t\times\nabla\tilde{\boldsymbol{f}}_t$ can be nonzero, since this is the source of equilibrium spin currents.

The final result shows that if one in equilibrium has a spin supercurrent $\boldsymbol{j}_{\mathrm{s}}^{\mathrm{eq}}$, then a nonequilibrium spin mode $\boldsymbol{h}_{\mathrm{s}}$ gives rise to a new component $\boldsymbol{j}_{\mathrm{s}}^{\mathrm{neq}} \sim \boldsymbol{j}_{\mathrm{s}}^{\mathrm{eq}}\times\boldsymbol{h}_{\mathrm{s}}$. This can intuitively be interpreted as the injected spins $\boldsymbol{h}_{\mathrm{s}}$ exerting some kind of torque on the spins transported by the equilibrium current $\boldsymbol{j}_{\mathrm{s}}^{\mathrm{eq}}$, thus producing a component $\boldsymbol{j}_{\mathrm{s}}^{\mathrm{neq}}$ that is spin-polarized in a direction perpendicular to both. This analogy is not perfect: it leaves out the Im and Re operations in Eq. (S86), and the fact that the cross-product relation is between *spectral* currents and accumulations. However, the intuition provided by this picture is sufficient to explain the results in the main manuscript.

III. NUMERICAL MODEL

As summarized in the main article, we perform the numerical calculations using the Usadel formalism [S1–S3, S13, S16]. This is formulated in terms of the 8×8 quasiclassical propagator

$$\check{g} \coloneqq \begin{pmatrix} \hat{g}^{\mathrm{R}} & \hat{g}^{\mathrm{K}} \\ 0 & \hat{g}^{\mathrm{A}} \end{pmatrix}, \tag{S87}$$

which satisfies the identities $\hat{g}^{\mathrm{K}} = \hat{g}^{\mathrm{R}}\hat{h} - \hat{h}\hat{g}^{\mathrm{A}}$ and $\hat{g}^{\mathrm{A}} = \hat{\tau}_3 \hat{g}^{\mathrm{R}\dagger}\hat{\tau}_3$. Together, these identities show that we have to determine two 4×4 matrices to know $\check{g}$: the retarded propagator $\hat{g}^{\mathrm{R}}$, which determines the spectral properties of a material; and the distribution function $\hat{h}$, which describes the occupation numbers of the states in the material. Both of these matrices can be functions of position $\boldsymbol{r}$ and quasiparticle energy ϵ.

In general, the distribution function $\hat{h}$ follows from solving a *kinetic equation* that can be derived directly from the full 8×8 Usadel equation. We present a complete derivation of a kinetic equation and relevant boundary conditions in Ref. S12, which is valid for quite general superconducting structures. The result is formulated as an explicit and linear differential equation, which can be easily and efficiently implemented in a numerical Usadel solver. Related derivations can be found in Refs. [S2, S3, S13, S17–S19]. However, instead of solving the kinetic equation explicitly, we have made two simplifying assumptions about the distribution function $\hat{h}$. The first is that it is roughly constant throughout the superconductor, which is reasonable as long as the superconductor is not too thick compared to its spin relaxation length. The second is that the distribution function can be modelled using a spin voltage, which we justify in the Discussion in the main article. These assumptions imply that we can treat $\hat{h}$ as a constant parameter, which simplifies our model system from a 2D to 1D geometry, thus making it much more feasible to attack numerically.

Since we treat $\hat{h}$ as a parameter, we only have to solve the Usadel equation for $\hat{g}^{\mathrm{R}}$. In an effectively 1D superconductor, such as the model system in the main article, this takes the form

$$i\xi^2 \partial_x(\hat{g}^{\mathrm{R}}\partial_x\hat{g}^{\mathrm{R}}) = [\hat{\Delta} + \epsilon\hat{\tau}_3,\, \hat{g}^{\mathrm{R}}]/\Delta_0, \tag{S88}$$

where $\hat{\Delta} = \mathrm{antidiag}(+\Delta, -\Delta, +\Delta^*, -\Delta^*)$ incorporates the superconducting order parameter $\Delta(x)$, $\hat{\tau}_3 = \mathrm{diag}(+1,+1,-1,-1)$ is a Pauli matrix in Nambu space, Δ_0 is the bulk zero-temperature order parameter, and ξ is the superconducting coherence length. In the numerical simulations, we also approximate the effect of inelastic scattering using a Dynes parameter: $\epsilon \mapsto \epsilon + 0.01 i\Delta_0$.

The ferromagnetic insulators in our model system were treated as spin-active interfaces, i.e. boundary conditions that account for spin-dependent phase shifts for quasiparticles in the superconductor that are reflected from the insulating interface [S21–S24]. This boundary condition takes the form

$$\pm 2L(\hat{g}^{\mathrm{R}}\partial_x\hat{g}^{\mathrm{R}}) = -i(G_\varphi/G_{\mathrm{N}})\,[\boldsymbol{m}\cdot\hat{\boldsymbol{\sigma}}, \hat{g}^{\mathrm{R}}], \tag{S89}$$

where the plus and minus signs describe boundary conditions at the left and right interfaces of the superconductor, respectively. Here, L is the length of the superconductor along the x-axis, G_{N} is the bulk conductance of the superconductor in its non-superconducting state, G_φ describes the effect of spin-dependent phase shifts when quasiparticles are reflected from a magnetic interface, and $\boldsymbol{m}$ is a unit vector that describes the magnetization direction at the same interface.

In addition to the equations above, we need to solve a self-consistency equation for the order parameter $\Delta(x)$ [S20],

$$\Delta(x) = \frac{1}{4\log(2\omega_c/\Delta_0)} \int_{-\omega_c}^{+\omega_c} \mathrm{d}\epsilon\; f_s^{\mathrm{K}}(x,\epsilon). \tag{S90}$$

Here, f_s^{K} is the singlet part of the anomalous part of the Keldysh propagator $\hat{g}^{\mathrm{K}}$. In practice, it can be evaluated at all energies from the calculated $\hat{g}^{\mathrm{K}}$ at positive energies using the identities

$$f_s^{\mathrm{K}}(x,+\epsilon) = \frac{1}{4}\,\mathrm{Tr}\left[(-i\hat{\sigma}_2)(\hat{\tau}_1 - i\hat{\tau}_2)\,\hat{g}^{\mathrm{K}}(x,+\epsilon)\right], \tag{S91}$$

$$f_s^{\mathrm{K}}(x,-\epsilon) = \frac{1}{4}\,\mathrm{Tr}\left[(+i\hat{\sigma}_2)(\hat{\tau}_1 + i\hat{\tau}_2)\,\hat{g}^{\mathrm{K}}(x,+\epsilon)\right]^*. \tag{S92}$$

These follow from parametrizing $\hat{g}^{\mathrm{K}}$ in a similar manner as Eq. (S75), then invoking the definition of tilde-conjugation $\tilde{f}_s(x,\epsilon) \coloneqq f_s^*(x,-\epsilon)$, and finally using standard trace identities.

The actual numerical implementation was done using the Riccati parametrization of the retarded propagator [S20, S25],

$$\hat{g}^{\mathrm{R}} = \begin{pmatrix} +N & 0 \\ 0 & -\tilde{N} \end{pmatrix} \begin{pmatrix} 1+\gamma\tilde{\gamma} & 2\gamma \\ 2\tilde{\gamma} & 1+\tilde{\gamma}\gamma \end{pmatrix}, \tag{S93}$$

where γ is a 2×2 complex matrix, and $N \coloneqq (1-\gamma\tilde{\gamma})^{-1}$. This matrix structure is defined in a way that automatically satisfies the normalization condition $(\hat{g}^{\mathrm{R}})^2 = 1$, and accounts for the particle–hole symmetries of the propagator, thus reducing the number of independent variables one has to solve for numerically. This parametrization also has the additional benefits that the Riccati parameter γ is single-valued and has a bounded norm, thus resulting in a more stable numerical solution procedure. How to reformulate the equations for $\hat{g}^{\mathrm{R}}$ in terms of γ is described in e.g. Ref. S20. In practice, we alternate between calculating γ from Eqs. (S88) and (S89), and updating $\Delta(x)$ using Eqs. (S90)–(S92), until they converge to a satisfactory degree. The simulation code itself is publicly available from github.com/jabirali/GENEUS.

[S1] J. Rammer and H. Smith, Rev. Mod. Phys. **58**, 323 (1986).

[S2] W. Belzig, F. K. Wilhelm, C. Bruder, G. Schön, and A. D. Zaikin, Superlattices and Microstructures **25**, 1251 (1999).

[S3] V. Chandrasekhar, in *Superconductivity* (Springer, Berlin, Heidelberg, 2008) pp. 279–313.

[S4] J. A. Ouassou, S. H. Jacobsen, and J. Linder, Phys. Rev. B **96**, 094505 (2017).

[S5] S. H. Jacobsen, I. Kulagina, and J. Linder, Sci. Rep. **6**, 23926 (2016).

[S6] N. B. Kopnin, *Theory of Nonequilibrium Superconductivity* (Clarendon Press, Oxford, United Kingdom, 2001).

[S7] I. V. Tokatly, Phys. Rev. B **96**, 060502 (2017).

[S8] S. H. Jacobsen, J. A. Ouassou, and J. Linder, Phys. Rev. B **92**, 024510 (2015).

[S9] J. P. Morten, *Spin and Charge Transport in Dirty Superconductors*, Master thesis, NTNU, Norway (2005).

[S10] F. S. Bergeret and I. V. Tokatly, Phys. Rev. Lett. **110**, 117003 (2013).

[S11] F. S. Bergeret and I. V. Tokatly, Phys. Rev. B **89**, 134517 (2014).

[S12] J. A. Ouassou, T. D. Vethaak, and J. Linder. Phys. Rev. B **98**, 144509 (2018).

[S13] F. S. Bergeret, M. Silaev, P. Virtanen, and T. T. Heikkilä. Rev. Mod. Phys. **90**, 041001 (2018).

[S14] G. E. W. Bauer, E. Saitoh, and B. J. van Wees, Nat. Mater. **11**, 391 (2012).

[S15] M. Eschrig, Rep. Prog. Phys. **78**, 104501 (2015).

[S16] K. D. Usadel, Phys. Rev. Lett. **25**, 507 (1970).

[S17] I. V. Bobkova and A. M. Bobkov, JETP Lett. **101**, 118 (2015).

[S18] M. Silaev, P. Virtanen, F. S. Bergeret, and T. T. Heikkilä, Phys. Rev. Lett. **114**, 167002 (2015).

[S19] F. Aikebaier, M. A. Silaev, and T. T. Heikkilä, Phys. Rev. B **98**, 024516 (2018).

[S20] S. H. Jacobsen, J. A. Ouassou, and J. Linder, Phys. Rev. B **92**, 024510 (2015).

[S21] M. Eschrig, A. Cottet, W. Belzig, and J. Linder, New J. Phys. **17**, 083037 (2015).

[S22] P. Machon, M. Eschrig, and W. Belzig, Phys. Rev. Lett. **110**, 047002 (2013).

[S23] A. Cottet, D. Huertas-Hernando, W. Belzig, and Y. V. Nazarov, Phys. Rev. B **80**, 184511 (2009).

[S24] A. Cottet, Phys. Rev. B **76**, 224505 (2007).

[S25] N. Schopohl, arXiv:cond-mat/9804064.

[S26] The fact that holes carry opposite spins from electrons can be seen from the field operators. In our formalism, a "spin-σ hole" refers to the quasiparticles counted by $\langle \Psi_\sigma \Psi_\sigma^\dagger \rangle$. This means that the creation operator for spin-up holes is the annihilation operator for spin-up electrons. Annihilating a spin-up electron *reduces* the spin in the system by $\hbar/2$, so a spin-up hole must actually carry net spin in the down-direction. In other words, a "spin-up hole" is "a hole that is left when a spin-up electron is removed", not "a hole that physically carries spin-up".
